# Determinants of Occupational Safety Culture in Hospitals and other Workplaces—Results from an Integrative Literature Review

**DOI:** 10.3390/ijerph17186588

**Published:** 2020-09-10

**Authors:** Anke Wagner, Ladina Schöne, Monika A. Rieger

**Affiliations:** Institute of Occupational and Social Medicine and Health Services Research, University Hospital of Tübingen, 72074 Tübingen, Germany; la.sch@t-online.de (L.S.); monika.rieger@med.uni-tuebingen.de (M.A.R.)

**Keywords:** integrative review, occupational safety culture, workplaces, hospital

## Abstract

Background: The aim of the present study was to obtain an overview of occupational safety culture by assessing and mapping determinants in different workplaces (hospital workplaces and workplaces in construction, manufacturing, and other industry sectors) using an already established theoretical framework with seven clusters developed by Cornelissen and colleagues. We further derived implications for further research on determinants of occupational safety culture for the hospital workplace by comparing the hospital workplace with other workplaces. Methods: We conducted an integrative literature review and searched systematically for studies in four research databases (PubMed, Web of Science, CINAHL, and PsycINFO). The search was undertaken in 2019, and updated in April 2020. Results of the included studies were analyzed and mapped to the seven clusters proposed by Cornelissen and colleagues. Results: After screening 5566 hits, 44 studies were included. Among these, 17 studies were conducted in hospital workplaces and 27 were performed in other workplaces. We identified various determinants of an occupational safety culture. Most studies in hospital and other workplaces included determinants referring to management and colleagues, to workplace characteristics and circumstances, and to employee characteristics. Only few determinants in the studies referred to other factors such as socio-economic factors or to content relating to climate and culture. Conclusions: The theoretical framework used was helpful in classifying various determinants from studies at different workplaces. By comparing and contrasting results of studies investigating determinants at the hospital workplace with those addressing other workplaces, it was possible to derive implications for further research, especially for the hospital sector. To date, many determinants for occupational safety culture known from workplaces outside of the healthcare system have not been addressed in studies covering hospital workplaces. For further studies in the hospital workplace, it may be promising to address determinants that have been less studied so far to gain a more comprehensive picture of important determinants of an occupational safety culture in the hospital sector.

## 1. Introduction

The promotion of occupational safety culture remains an important issue in various workplaces. Previous studies, mainly conducted in the industrial sector, identified several determinants and predictors that promote an occupational safety culture. Zohar (2011) addressed different antecedents of safety climate, and based on these, developed a conceptual model [1]. According to Zohar, the following seven antecedent variables shape a good safety climate: structural attributes of the work environment, symbolic social interaction, group and organization leadership, psychological work ownership, organizational commitment, job stress, burnout, and personality [1].

One recent quantitative review by He et al. [2] extended Zohar’s conceptual model by providing a quantitative overview on different antecedents and factors of safety climate. The identified antecedents were grouped into three main categories: situational factors (e.g., job and organizational characteristics, leadership, co-worker influence), interpersonal interactions (e.g., leader-member exchange, team-member exchange), and personal factors (e.g., personality characteristics, demographics) [2]. The authors calculated effect sizes for 38 antecedents to determine the magnitude of each within the three categories [2]. The authors detected the strongest effect sizes and associations for safety climate in particular for antecedents of interpersonal interactions, and situational factors [2]. As stated by He et al., there is an ongoing need for research on further antecedents and determinants of safety culture [2].

Another study by Beus et al. combined different theories about workplace safety in one integrated safety model and evaluated components of the model in the context of a systematic literature search [3]. The hereby developed model distinguished between distal (e.g., individual differences, contextual factors, job characteristics) and proximal antecedents (e.g., personal resources, safety knowledge, skills, or motivation) of safety-related behaviors and subsequent accidents and accident rates across individual and group levels of analysis, and suggested future research activities [3]. The authors found strong empirical support for the linkage between work behaviour and accidents, and for example weak empirical support for the linkage between individual differences (attitudes, abilities) and safety knowledge, skills and motivation [3]. According to previous work by Clarke (2010), some dimensions of psychological climate (job, role, group, leader, and organizational attributes) are also important antecedents and predictors of safety climate [4].

One of the most comprehensive and detailed overviews on determinants in literature on occupational safety is demonstrated by Cornelissen and colleagues [5]. The authors identified and clustered possible determinants that support occupational safety of employees in the following high-risk industries: construction, petro-chemistry, warehouses, and manufacturing [5]. In their study, they categorized the identified factors into seven clusters: Workplace characteristics and circumstances (cluster 1), Climate and culture (cluster 2), Management and colleagues (cluster 3), Employee characteristics (cluster 4), External (cluster 5), Performance (cluster 6), and Safety outcomes (cluster 7) [5]. Table 1 shows a detailed description of each cluster with the corresponding topics and categories.

In healthcare, and especially in the hospital workplace, meanwhile, there is a variety of studies addressing safety culture. However, in many studies, safety culture refers only to patient safety culture [6,7], and occupational safety culture of employees is not addressed. In some studies, occupational safety culture was considered in addition to patient safety culture [8,9,10,11,12,13,14], but did not represent the main aspect. The role of working conditions with regard to work-related injuries in healthcare (e.g., needle stick injuries) has been widely studied [15,16]. Similarly, the general relationship between safety culture and work-related injuries in healthcare has been well documented [14,17]. Employees in healthcare, and particularly in hospital workplaces, are confronted with high demands in their daily working conditions. Besides high workload, staff shortage, and shift working, employees have to deal with suffering and dying patients and their families, time pressure, perceived lack of reward, and sometimes conflict with other professions [18]. For employees in these professions, there is both a physical as well as a continuous psychological burden, which can have an impact on safety culture.

However, determinants of a comprehensive occupational safety culture have not often been described or categorized in contrast to other workplaces. Furthermore, at the current time, findings on determinants of occupational safety culture from other workplaces are seldom discussed to develop and promote an occupational safety culture in hospitals. In our opinion, it can be very useful, in particular for the development and promotion of an occupational safety culture, to include the experiences of other workplaces. In recent years, a lot of research on occupational safety culture has taken place, especially in the area of construction [19], and manufacturing [20]. Therefore, knowledge from these work areas may be useful to further promote occupational safety culture in hospitals. We therefore conducted an integrative review and focused on the following two research questions:

1. What are possible determinants of occupational safety culture from the perspective of employees in different workplaces (hospital, construction, manufacturing, and other industry sectors)?
2. What implications for further research on determinants of occupational safety culture for the hospital workplace can be derived by comparing the hospital workplace with other workplaces?

We pursued the objective to build a link between hospital workplaces and other workplaces by generating an overview of determinants of occupational safety culture in different workplaces. The obtained overview and the comparison of determinants in different workplaces can be helpful in identifying possible research requirements and implications, especially for hospital workplaces. The seven clusters from Cornelissen et al. [5] were thereby considered as a suitable framework to identify, summarize, and classify possible determinants in different workplaces since the authors considered quantitative and qualitative studies [5], and included determinants (e.g., external factors), which have received so far little attention in previous models.

## 2. Materials and Methods

The integrative review was carried out according to the procedure described by Whittemore and Knafl [21]. According to Whittemore and Knafl, an integrative literature review comprises the main methodical steps: (1) literature search, (2) data evaluation, and (3) data analysis [21]. We followed and adapted the PRISMA checklist (Preferred Reporting Items for Systematic reviews and Meta-Analyses) for the current review [22] (Supplementary Material, Table S1).

### 2.1. Literature Search

#### 2.1.1. Search Strategy

The aim for the search strategy was to perform a highly specific search by combining the central terms (using the Boolean operators “AND” and “OR”). We developed a search strategy for one database (Pubmed) and combined different terms for safety culture (e.g., safety culture, culture of safety, safety climate, prevention culture, organizational culture or climate) occupational safety (e.g., occupational health, occupational safety or occupational health and safety, industrial safety, job-safety, working safety or safety at work), and workplaces (e.g., workplace, working condition, work environment, hospital) using “AND”. We used different strategies and developed a text word search for each database, and if possible, a MeSH term or thesaurus term search. The search strategy was subdivided according to the setting: One search strategy followed studies in the hospital setting; the other search strategy was directed at studies conducted in other workplaces. For quality assurance reasons, the search strategy was evaluated by two different persons (A.S. and M.A.R.) based on the PRESS Guideline [23]. After feedback, the search strategy was revised, finalized, and then transferred (if necessary, including translation) to three other databases (Web of Science, PsycINFO and CINAHL). The final search strategy for the database Pubmed can be viewed in the Supplementary Material (Table S2 and Table S3).

#### 2.1.2. Inclusion and Exclusion Criteria

We used the SPIDER-Framework [24] to determine inclusion and exclusion criteria with regard to the four dimensions: Sample, Phenomenon of Interest, Design, Evaluation, and Research type. We also considered the inclusion and exclusion criteria of the systematic literature review conducted by Cornelissen and colleagues for orientation [5]:

- “Sample”: The targeted sample comprised employees at different workplaces. Investigated workplaces were hospital workplaces and other workplaces (e.g., construction, manufacturing, services, and other industry sectors). We excluded studies conducted in some countries (Israel, Iran, Africa, Chile and Korea) [25,26,27,28,29,30,31,32,33,34,35], due to the difficulties to compare the results to the German healthcare system. Furthermore, we excluded studies at nuclear power stations or in the oil and gas industry since the comparison with workplaces in construction and manufacturing was of primary interest to us. In addition, the nuclear power and gas industries often focus on the prevention of accidents/serious incidents, whereas we were interested in the occupational safety culture “in everyday life”.
- “Phenomenon of Interest”: We included studies that described different determinants of an occupational safety culture. We excluded studies in which occupational safety culture at the workplace was not the main aspect.
- “Design”: We intended to consider studies with different research methods (questionnaire, qualitative interviews, focus group discussions).
- “Evaluation”: Studies of interest included the perceptions and experiences of employees on occupational safety culture. We used the seven clusters from Cornelissen et al. as a raster to evaluate and sort the determinants that were assessed in the individual studies.
- “Research type”: We included studies with different research designs (qualitative, quantitative, mixed-method) aiming to gain a more comprehensive view on utilized determinants of an occupational safety culture in different workplaces. We excluded intervention studies on occupational safety culture or on occupational safety as we did not want to evaluate the effects of individual interventions. Our interest was focused on the determinants identified in the studies.

For our study, we used a rather broad definition of safety culture to find sufficient hits in the literature search. Therefore, we did not distinguish between the two concepts of safety culture and safety climate. The studies had to be published in peer-reviewed journals since the year 2000 to cover the last 15 years, and to reflect potential changes in the way occupational safety culture is seen at different workplaces. The studies had to be published in German or English language.

#### 2.1.3. Literature Search

We searched in four databases (Pubmed, PsycINFO, CINAHL, and Web of Science) to identify relevant literature for our research aim. The search was conducted in February and March 2019 (last search was carried out on March 21, 2019) and updated in April 2020 (last search performed on April 04, 2020). In addition to the database search, the reference list of selected publications was considered. Furthermore, the following websites were searched for further literature: OSHA (Occupational Safety and Health Administration), DGUV, BAuA, and INQA.

#### 2.1.4. Screening and Selection Procedure

Two raters (A.W. and L.S.) screened all references independently. In the screening process, we included studies that examined safety culture and occupational health and safety. The screening was conducted using the Rayyan program [36]. At the stage of the full text analysis, we included studies that described or identified possible determinants of safety culture in the workplace. In case of disagreement between the two reviewers, there was always the possibility to call in a third person (M.A.R.). Disagreement between the two reviewers was resolved by consensus discussion.

### 2.2. Data Evaluation

#### 2.2.1. Critical Appraisal

A quality appraisal for each selected study was conducted in the next step. We used the SURE Checklist for Cross-sectional studies (12 items) [37], and for longitudinal studies, we employed the SURE Checklist for Cohort studies (13 items) [38]. In both checklists, all single quality rating items were answered with “yes”, “can’t tell”, or “no”. For mixed-methods studies, we applied the Mixed Methods Appraisal Tool (MMAT), which included five questions and also the possibility to answer with “yes”, “can’t tell”, or “no” [39]. All quality rating items are shown in the Supplementary Material (see Table S4). For each study, we calculated how often items from the checklist were answered with “yes” in percent and considered values above 60% as satisfying quality (see Table S5 in Supplementary Material).

#### 2.2.2. Data Analysis

We followed the procedure described by Whittemore and Knafl for data analysis with data reduction, data display, and data comparison [21]. For data analysis, we developed a data sheet and extracted study characteristics from the included studies (see Table S5 in Supplementary Material). We investigated the variables of the individual studies and categorized them according to each of the seven clusters of Cornelissen et al. using a concept mapping strategy [40]. The detailed mapping of the individual variables is shown in Table S5 in Supplementary Material. After mapping, we calculated how often the clusters are represented in the different workplaces (hospital versus other workplaces) to identify possible research gaps (see Table 2 and Table 3).

## 3. Results

### 3.1. Summary of Search Results

**Figure 1:**
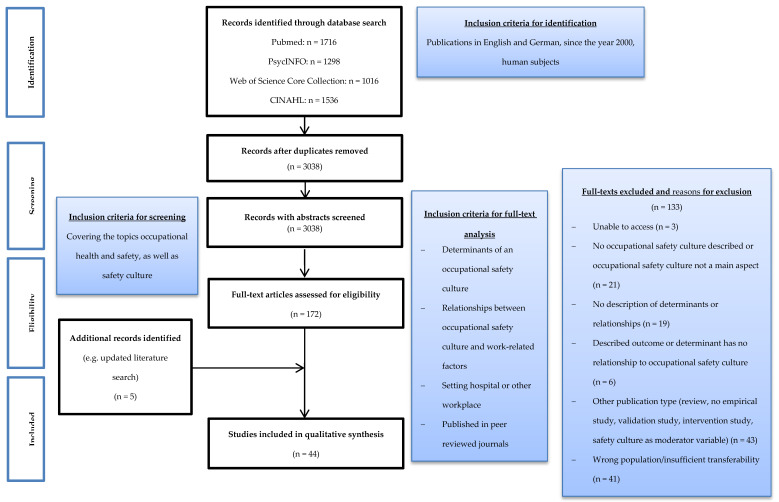
Study selection.

The literature search yielded 5566 hits. After removing duplicates, 3038 results remained for title and abstract screening. Following the screening, we included 172 publications in the full text analysis. After the full text analysis and critical appraisal, a total of 44 studies were included in the literature review. The selection process of the studies is outlined in Figure 1.

### 3.2. Characteristics of Included Studies.

We identified 44 studies investigating various determinants of an occupational safety culture [41,42,43,44,45,46,47,48,49,50,51,52,53,54,55,56,57,58,59,60,61,62,63,64,65,66,67,68,69,70,71,72,73,74,75,76,77,78,79,80,81,82,83,84]. Seventeen studies referred to hospital workplaces [41,44,48,54,56,62,63,64,65,66,67,69,70,71,80,81,84] and 27 studies were conducted at other workplaces, mostly manufacturing, construction, and other industry sectors [42,43,45,46,47,49,50,51,52,53,55,57,58,59,60,61,68,72,73,74,75,76,77,78,79,82,83].

The 44 studies were published between 2000 and 2020. Most of the studies applied a cross-sectional research design [41,42,43,44,45,46,47,48,49,50,51,52,53,54,55,57,58,59,60,61,62,63,64,65,68,69,71,72,73,74,75,76,77,78,79,80,81,83,84]. We identified four cohort studies using a longitudinal research design [56,67,70,82], and only one study employing a mixed-methods design [66]. Fifteen studies were conducted in European countries: five in Spain [51,52,53,73,83], three in Sweden [61,72,82], two in Italy [43,48], one in the Netherlands [44], one in Austria [45], one in the United Kingdom [47], one in Serbia [68], and one in Portugal [78]. Seventeen studies were carried out in the United States of America (USA) [41,49,50,54,56,58,59,60,62,71,74,75,76,77,79,80,81]. We identified three studies conducted in Canada [46,63,64], seven studies conducted in Australia [55,57,66,67,69,70,84], and one cross-national study referred to research undertaken in the United States of America and in Canada [65]. One study did not specify where the research was carried out, but only stated that the results referred to 19 countries [42].

Most of the studies used self-report questionnaires to capture safety culture. In some studies, the questionnaires were combined with other gathered data (e.g., routine data, injury reports, injury database, safety audit) [41,56,59,67,70,75,76,78,81,84]. One mixed-methods study used both a scale from a questionnaire and qualitative interview data [66]. The conducted quality appraisal revealed satisfying quality values above 60% for most of the studies. Some studies achieved values under 60% [44,48,55,67,69,71,74,75,77]. The main reasons for the negative appraisals were lack of information on eligibility and on selection of the study participants. A comprehensive overview of the studies is presented in the Supplementary Material (Table S5).

The overview of how frequently and in which clusters determinants from the individual studies were represented is shown with regard to hospital (Table 2) and other workplaces (Table 3). Neither for hospital workplaces nor for other workplaces were all clusters addressed simultaneously by at least one study. Instead, most of the studies reported on determinants attributed to the cluster “Management and colleagues” (16/17 studies on hospital workplaces, 25/27 studies on other workplaces), followed by “Employee Characteristics” (14/17 (hospitals) and 23/27 (other), respectively), and “Workplace characteristics and circumstances” (14/17 studies on hospitals and 21/27 studies on other workplaces, respectively). The least frequently investigated determinants belonged to the clusters “External (Factors]” (0/17 studies on hospital workplaces and 2/27 studies on other workplaces) and “Climate and Culture” (3/17 on hospital workplaces and 1/27 on other workplaces, respectively).

### 3.3. Determinants of An Occupational Safety Culture

#### 3.3.1. Cluster “Workplace Characteristics and Circumstances”

Fourteen out of the 17 studies in hospital workplaces addressed “Workplace characteristics and circumstances” (see Table 2). Studies in this cluster covered the following topics: accessibility, availability, and quantity of safety equipment [54]; exposure to workplace hazards and risks [54,71,80,81]; perceived workplace or job safety [63,64,65]; and description of work and hospital characteristics (e.g., work arrangement, work role and position, workload, job stress, role clarity, patient/client contact, and patient care rates, hours worked, number of workers in the team, hospital status) [41,44,54,62,63,64,66,69,80,81,84]. Some individual data, like union membership [80], work engagement [67], and employment status [64,84], were also addressed. Two studies mentioned aspects of psychosocial working conditions (e.g., conflict with others, lack of support, emotional demands, bullying, skills discretion) [62,67].

In 21/27 of the studies in other workplaces, topics from the cluster “Workplace characteristics and circumstances” were also included (see Table 3). Common topics were safety-related issues like average working environment risk level or exposure to common workplace hazards [42,68,73,78,83], availability and safety conditions of safety equipment and machinery [53,78], promotion of overall health and well-being [60], and perceived safety or health at work [49,50,59,60,72,75,78]. Some studies also focused on descriptions of workplace characteristics and covered the following topics: type of organization or department [43,68,72,73,78], OHSAS 18001 (Occupational Health and Safety Assessment Series) certification [72], geographical location of the organization [57], and company size or number of employees [45,46,53,72,73]. Other topics included information on employees, like kind of job contract or type of employment [43,53,57,73], respondents’ role or job position [45,46,47,53,57,60,68,72,73,78], work shift [43], weekly working hours or number of hours worked [46,49,60,72], and individual data like union membership [46]. Some studies addressed organizational environment and functionality [68,72], environmental or physical working conditions [49,57,72], the quality of environmental working conditions (e.g., humidity, lighting, ventilation, temperature, workspace) [49,73,83], and work-related safety practices [74]. Other studies focused on different aspects of psychosocial working conditions [42,45,46,52,53,61,72,74,75,82]. One study mentioned specific work limitations (e.g., physical demands, time management) [60].

#### 3.3.2. Cluster “Climate and Culture”

Only 3/17 of the studies in hospital workplaces mentioned “Climate and culture” aspects (see Table 2). We identified three studies referring to organizational climate [48,69,81]. One study from the hospital sector reported the following topics: affective, cognitive, and instrumental factors of organizational climate [48]. The affective factor included aspects of social and interpersonal relationships between employees [48]. The cognitive factor comprised perceptions related to psychological involvement in the workplace, and the instrumental factor consisted of structural aspects [48]. Further topics within this cluster were general organizational climate with specific aspects of the work environment (e.g., appraisal and recognition, goal congruency, participative decision-making, professional growth, professional interaction) [69], and perceived organizational climate [81].

Only 1/27 of the studies from other workplaces included topics that could be assigned to the cluster “Climate and culture” (see Table 3).

#### 3.3.3. Cluster “Management and Colleagues”

Most studies in hospital workplaces (16/17) addressed topics from the cluster “Management and colleagues” (see Table 2). Topics can be assigned to management (e.g., management priority given to health and safety; management commitment to health and safety, manager values, manager support…) [44,48,54,62,63,64,65,66,67,69,70,80,84], to supervisors (e.g., supervisor safety, supervisor support, supervisor safety leadership…) [41,56,63,64,65], and to co-workers (e.g., co-worker influence, group-norms, co-worker safety, and co-worker support) [44,63,64,65]. On the other hand, some of the studies in hospital workplaces included themes that referred to the management of occupational safety, e.g., organizational communication and feedback about safety issues [44,54,62,66,67,69,80,84], organizational participation [44,66,67,84], and the implementation of different safety systems and safety procedures (safety precautions, safety trainings, safety workarounds and safety programs) [54,62,63,64,65,69,71,80].

Similar to studies from hospital workplaces, topics from the cluster “Management and colleagues” were also addressed in 25/27 of the studies from other workplaces (see Table 3). Common topics for management were: safety communication [43,45,49,51,52,55,57,59,68,72,73,75,76,77,78,82,83], safety training or safety practices [43,45,46,51,53,55,58,68,72,75,77], safety rules, safety standards or safety policies and programs [49,50,51,53,57,68,72,73], safety management and leadership [57,72,73,83], management values [43,45,55,75,77], management commitment to safety and competence, managers’ attitudes towards safety, and managers’ behaviour towards safety [46,51,52,58,59,72,74,79,82]. Safety inspections [42,55], improvement of safety systems and continuous improvements [43,72,75,77,78], priority and importance of safety issues within the organization [49,50,53,79,82], and organizational or management support for safety [49,50,68] were also included. Specific topics in individual studies comprised return-to-work policies [58], accident or risk prevention [53,68], post-injury administration [58], deployment of safety delegates [53,72], occupational health services [72], top management safety empowerment, and safety justice [79]. Other topics that affect management are: management reaction and investment [53,68,78], planning and control activities [51,53,72]. Finally, the following topics were also addressed: specific and different leader influence tactics [47,72], and incentives [51,52]. Common topics for supervisors were: safety communication [42], supervisor’s reaction to workers’ behaviours [43], supervisor’s effort to improve safety [42,43,53], supervisor safety perception [46], supervisory action and expectation [42,47,53], supervisor enforcement of safety policies [42,77], supervisor concerns related to workers’ safety practices [78], supervisor support [74], supervisor safety priority, commitment and competence, and supervisor safety empowerment and justice [79]. Common topics for co-workers were: safety communication [43], safety mentoring [43], safety systems [43], co-workers values [43], co-worker safety perception [46], co-worker support [49], and co-worker safety commitment [79].

#### 3.3.4. Cluster “Employee Characteristics”

Fourteen of 17 of the studies in hospital workplaces included employee characteristics (see Table 2). The most common topics were age [41,44,54,64,65,66,80,81,84], gender [41,44,54,65,80,84], years of experience [64,65,81], tenure or length of employment [41,44,66,80], and educational level [54,64,65,80,81]. In addition to safety culture, further studies in hospital workplaces assessed job satisfaction [64,65,71], turnover intentions [64,65,71], self-rated health status [65,67], and lifestyle habits (e.g., smoking, exercise) [44]. Some studies also recorded negative affect [70,71], race, ethnicity, and social background information [80].

Twenty-three of 27 of the studies conducted at other workplaces also addressed employee characteristics (see Table 3). Similar to the studies in hospital workplaces, the most common topics were age [42,43,46,47,49,53,57,60,68,72,73,78], gender [43,46,47,49,53,57,60,68,72,73,78], educational level [43,53,60,68,73], and length of employment or work experience and organizational tenure [42,43,46,47,49,53,68,78]. Further studies also included specific safety characteristics (e.g., safety motivation, safety knowledge, safety awareness and competency, hazards recognition, safety control, previous involvement in work accidents, and individual responsibility) [45,46,51,54,55,58,59,61,68,72,73,74,75,78,83]. Other factors within this cluster were assessments of individual resilience [46], organizational trust [59,78], employee satisfaction [52,59,60,61], lateness [50], and turnover intention [50,52,59]. Additionally, self-rated health (e.g., vitality) [50,60], lifestyle behaviours (tobacco, alcohol, emotional or physical abuse, physical activity, nutrition, sleep) [60], and other socio-demographic data were collected (e.g., nationality, children) [43,53].

#### 3.3.5. Cluster “External (Factors)”

We were unable to identify any studies in hospital workplaces which addressed topics belonging to the cluster “External (factors)”.

Only 2/27 of the studies conducted in other workplaces addressed aspects from the cluster “External (factors)” (see Table 3). Two studies included customer satisfaction as part of firm competitiveness and the existence of a budget for occupational health and safety management as part of occupational health and safety management [52,72].

#### 3.3.6. Cluster “Performance”

Eight of 17 of the studies in hospital workplaces included topics that could be assigned to the cluster “Performance” (see Table 2). The studies addressed safety compliance [41,48,54,56,62,69,70,71] and safety participation of the employees [48,62,69,70].

Nineteen of 27 of the studies from other workplaces included topics from the cluster “Performance” (see Table 3). The most common topics were also safety compliance and adherence [43,45,52,53,55,57,75,79,82], and safety participation of the employees [43,45,47,49,52,55,79]. Other topics within this cluster were safety involvement and safety-specific behaviour (e.g., suggestions and reports to supervisors, using available safety protection equipment, structural safety behaviour, interactive safety behaviour, personal safety behaviour, choosing safe working methods and procedures, taking no shortcuts with safety, prioritizing safety, workers’ commitment to safety, organizational commitment, risk acceptance, and safety audit) [45,50,51,53,55,57,61,72,73,78,82,83]. Three studies dealt with aspects of organizational performance within this cluster. Two studies included aspects of production performance (e.g., product quality, productivity, image and reputation, and innovation) [52,60], and another study employed aspects of financial performance (e.g., company profitability, solvency, and creditworthiness [72].

#### 3.3.7. Cluster “Safety Outcomes”

Twelve of 17 of the studies in hospital workplaces included topics related to the cluster “Safety outcomes” (see Table 2). Topics were injuries [41,56,64,65,71,81,84], safety incidents and accidents [54,66,67,70], reporting and underreporting of injuries [56,84]. Sick days, illnesses, physical and psychosocial disorders (e.g., musculoskeletal disorders, emotional exhaustion, burnout), days missed from work, and other factors (e.g., absenteeism; presenteeism; healthcare utilization) were also addressed [41,44,64,67,84].

Topics from the cluster “Safety outcomes” were included in 16/27 of the studies from other workplaces (see Table 3). Analogously to the studies in hospital workplaces, common topics were reported, such as injuries [51,52,58,59,75] and safety incidents and accidents [46,50,68,72,75]. One study distinguished between safety incidents with regard to expected frequency and expected severity [74]. Some studies also addressed topics like reporting and underreporting of injuries and accidents, and provided information about reported and unreported injuries and accident rates [76,77]. Two studies classified work-related accident rates into four categories: number of near misses, minor accidents, accidents resulting in up to 3 days off work, and severe accidents resulting in more than 3 days off work [73,83]. Another study also addressed work time missed because of health-related and non-health-related issues [60]. Three studies mentioned consequences from safety incidents, like physical and psychological stress symptoms (e.g., emotional exhaustion) [46,57] and disorders (e.g., back pain, depression) [60]. Three studies also communicated other safety-related outcomes (e.g., absenteeism from work and material damage) [50,51,52].

#### 3.3.8. Classification of the Studies into the Topics and Categories according to Cornelissen et al.

We compared our findings with topics and categories identified by Cornelissen et al. [5]. Table 4 summarizes for each cluster which of the studies addressed the respective topics and categories as proposed by Cornelissen et al. [5].

## 4. Discussion

We conducted an integrative literature review to assess and map determinants of an occupational safety culture in different workplaces (hospitals and workplaces in construction, manufacturing, warehouses, and others) using the seven clusters described by Cornelissen et al. [5] as a framework.

The obtained overview and the comparison of determinants in different workplaces facilitate the identification of possible research requirements and implications, especially for hospital workplaces. We discuss the results for each of the seven clusters below, and compare our findings with antecedents identified in other models [2,3,4].

### 4.1. Determinants of An Occupational Safety Culture

#### 4.1.1. Cluster “Workplace Characteristics and Circumstances”

The topics addressed within this cluster were almost used equally by the studies at different workplaces (14/17 at hospital workplaces versus 21/27 at other workplaces). In general, we found a variety of different determinants. This cluster represented also determinants mentioned in other models, like job demands or job role [2,4]. Compared with topics and categories from Cornelissen et al. [5], we found determinants for physical work environment, work characteristics, and workforce. Most determinants were addressed in the category work characteristics (see Table 4). This may be because the included studies in the different workplaces mainly considered the perspective of employees and how employees perceive their workplace, for example. Therefore, other characteristics such as workforce quantity and workforce composition were covered less in the studies. Generally, studies in hospital workplaces revealed fewer determinants in this cluster compared to studies from other workplaces. In particular, physical and psychosocial working conditions were not recorded to the same extent. In addition, new and emerging occupational safety and health risks associated with digitalization like increasing work stress and ergonomic risks [85] were also not addressed in the studies included in this review. For future studies in hospital workplaces, it may be promising to include and address further determinants from this cluster, and to cover aspects from physical work environment, (changing) work characteristics, and workforce. It also seems promising to address further topics associated with digitalization since the emergence of digitalization affects more and more employees in their workplaces [85].

#### 4.1.2. Cluster “Climate and Culture”

Overall, this cluster was not very well represented in the studies on hospital workplaces (3/17) or other workplaces (1/27). In our opinion, it is not completely clear why this cluster is so rarely represented in studies. One reason is certainly that the topics in this cluster were not further differentiated, but were only divided into organizational culture/climate and safety culture/climate. This complicated the assignment of content within the mapping. Another explanation could be that the included studies mainly considered the perspective of employees and not the perspective of management and supervisors. The evaluation of organizational safety culture and climate aspects may be better captured in studies that surveyed supervisors and management besides employees. Another explanation may be that topics from this cluster were not often included in questionnaires. In many cases, safety culture questionnaires focus on specific and easily detectable topics, such as leadership, safety behaviour or safety outcomes, and other themes are not assessed. Other models also included topics from this cluster [2]. He et al. named organizational culture as one facet of organization characteristics and as a subtopic from situational factors [2]. The study by He et al. found a strong association between organizational climate and safety climate. One implication for future surveys is certainly to use more assessment instruments that measure these topics in order to gain more insights and to address comprehensively the content of this cluster.

#### 4.1.3. Cluster “Management and Colleagues”

Topics from the cluster “Management and colleagues” were most represented among the studies included in this review. Sixteen of 17 of the studies in hospital workplaces and 25/27 from other workplaces were related to the cluster “Management and colleagues”. Compared with topics and categories by Cornelissen et al. [5], we identified determinants for management attitudes and behaviours, for co-worker attitudes and behaviours, and also for management of safety. It was not surprising that management and supervisors played an important role regarding safety culture in all identified studies. Other studies also confirmed the important role of management, supervisors, and co-workers in shaping safety culture [86]. Christian et al. performed a meta-analysis about workplace safety and confirmed that leadership constituted an integral component for improved workplace safety in addition to other person and situation-related factors [87]. The relevance of leadership and the influence of co-workers is also addressed in other models. He et al. was also able to show in his quantitative overview moderate to strong associations of leadership and co-workers with safety climate [2]. Thus, leadership and co-workers are important determinants of an occupational safety culture.

#### 4.1.4. Cluster “Employee Characteristics”

Another frequently discussed cluster was “Employee characteristics”. The topics addressed within this cluster were used equally by the studies addressing hospital (14/17) and other workplaces (23/27). Most of the studies assessed demographic aspects of the employees like age, gender or education. Specific career and job attitudes (e.g., tenure, job satisfaction, trust) and some lifestyle habits were also captured. In contrast to hospital workplaces, studies at other workplaces addressed specific safety characteristics (e.g., safety motivation, knowledge, awareness and competency…) to a larger extent. He et al. did not mention safety characteristics in his overview although previous work indicated the important role of safety knowledge and safety motivation [3,87]. Also, lifestyle habits were rarely listed as determinants in the studies included in the overview [2]. It seems useful to reflect safety characteristics and lifestyle habits in hospital workplaces as well, and future studies should address these aspects.

#### 4.1.5. Cluster “External (Factors)”

Topics from the cluster “External (Factors)” were given the least attention. We found no studies that included determinants from this cluster for hospital workplaces, and identified only 2/27 for other workplaces. The determinants identified according to Cornelissen et al. were only related to stakeholders (customer satisfaction) and to socio-economic issues (budget for occupational health and safety management). We found no studies that addressed aspects of governmental bodies, for example. This finding is supported by Cornelissen et al. [5], who stated that this cluster is rarely represented in other workplaces and that there is a gap in research regarding this cluster [5]. We did not find other published models who discussed these topics either.

#### 4.1.6. Cluster “Performance”

The cluster “Performance” was represented differently in the various workplaces. Only 8/17 of the topics from this cluster referred to hospital workplaces. In contrast, 19/27 of the studies at other workplaces included topics that could be assigned to this cluster. In hospital workplaces, the topics referred only to safety-related performance, like safety compliance and/or safety participation. No other topics were addressed. At other workplaces, most of the topics also concentrated on safety compliance and/or safety participation. However, we identified three studies that included topics related to organizational performance. This uneven distribution is in line with findings by Cornelissen et al. [5]. The authors also found that safety-related performance aspects were more addressed in their study than aspects of organizational performance [5]. In other models, performance and in particular safety-related work behavior is a common topic of discussion [3]. However, there is no difference between safety-related or organizational performance, and topics from organizational performance were seldom discussed. In general, studies in hospital workplaces should integrate more topics from the cluster “Performance”, and possibly also aspects of organizational performance.

#### 4.1.7. Cluster “Safety Outcomes”

This cluster was mentioned more often in hospital workplaces than in other workplaces (12/17 versus 16/27). Compared with topics and categories by Cornelissen et al., the studies included in the current study covered work-related incidents, accidents, and injuries in employees. We also assigned other topics into this cluster, like physical and psychological or psychosomatic disorders, although these topics are not mentioned in this cluster by Cornelissen et al. In our opinion, this cluster plays an important role for safety culture in hospitals. Hospital workers are generally at a high risk of getting injured (e.g., needle stick injury) or suffering an accident at work (e.g., heavy lifting while transferring patients). Another reason why this cluster is well represented in studies in the hospital setting may be the now well-established patient safety reporting system. In many countries, it is now standard to monitor and analyze (patient) safety outcomes carefully. We therefore assume that many studies in this area particularly consider safety outcomes as essential when it is necessary to assess occupational safety culture. As already mentioned, the relationship between safety culture and safety outcomes is well-documented in healthcare research [8,81], and future studies should comprehensively consider topics from this cluster. However, in our opinion, this cluster needed to be broadened in order to be able to address topics such as physical, psychological, and psychosomatic disorders. In addition, the reporting and underreporting of safety outcomes seems to be an important issue that should be addressed to a larger extent in further studies in the hospital sector.

### 4.2. Summary and Recommendations for Future Studies

We identified and mapped different determinants of occupational safety culture in various workplaces (hospital and workplaces in construction, manufacturing, and other industry sectors) using the seven clusters described by Cornelissen et al. [5]. As indicated in Table 2 and Table 3, we were unable to find a study in the different workplaces that covered determinants in all seven clusters. This raises the question of whether future studies should cover determinants from as many clusters as possible. Our overview shows that, in particular, determinants in the clusters “Climate and Culture” and “External (Factors)” have received little consideration in previous studies, so far. For future studies, it may be promising to include determinants from these two clusters. The integration of further factors can help to discover relationships between further determinants and to broaden perspectives on occupational safety culture. The overview also revealed that the same determinants of occupational safety culture are often measured in studies at different workplaces. We did not find any major differences regarding the investigated determinants in different workplaces. It may be helpful for future studies in the hospital sector to (1) consider the findings of studies on occupational safety culture in other workplaces, and (2) specifically select determinants and investigate them in the hospital sector. This may help to understand occupational safety culture in hospital workplaces more comprehensively.

### 4.3. Strengths and Limitations of the Study

The use of the seven clusters from Cornelissen et al. to classify and map possible determinants from different workplaces was suitable and helpful in comparing studies assessing hospital workplaces and other workplaces. Based on the conducted concept mapping of different determinants and the standardized comparison of studies reporting on different workplaces, we gained insights into the priorities in previous research on occupational safety culture and which aspects deserve future priorities. In addition to the imbalance in the assessment of determinants covering the seven individual clusters, new and emerging occupational health and safety risks associated with digitalization should be presented, e.g., in surveys or observations of work processes.

This review also had some methodological limitations. We conducted an integrative literature review with highly heterogeneous studies and diverse definitions of safety culture and safety climate. The concept mapping of possible determinants was carried out based on the information in the studies. In rare cases, the information lacked details, e.g., some studies did not provide the complete questionnaire with scales and items. In addition, it can be assumed that certain variables are only published in internal reports. For future research work, it might be useful to include internal reports or results from industry sector surveys or similar approaches in hospitals in order to get a more comprehensive picture of different determinants of occupational safety culture. Another limitation lies in the exclusion criteria. We excluded some countries due to a poor comparability to the working situations in Germany, and focused only on specific workplaces (mainly hospital, construction, manufacturing, and other industry sectors) and on the perspectives of employees. Also, the inclusion of studies only published in English or German should be considered as a limitation. It is possible that including studies from more countries, other workplaces, and the perspectives of supervisors and management would have resulted in more hits and more detected possible determinants in the seven clusters.

## 5. Conclusions

The seven clusters by Cornelissen et al. were useful in classifying various determinants from studies at different workplaces. By comparing and contrasting previously investigated determinants at the hospital workplace with other workplaces, it was possible to derive implications for further research, especially for the hospital sector. Comparing different economic sectors, many determinants identified from workplaces in e.g., construction work have not been addressed to the same extent in studies covering hospital workplaces, to date. In particular, specific topics from Cluster 2 according to Cornelissen et al. (e.g., safety climate/culture), Cluster 4 (e.g., safety characteristics, lifestyle habits), Cluster 5 (e.g., external factors), Cluster 6 (e.g., organizational performance), and Cluster 7 (e.g., consideration of other outcomes such as physical and psychosomatic disorders, reporting and underreporting of injuries) have been given little attention in hospital workplaces so far and should be included in further studies. It may be promising for future studies on hospital workplaces to assess these topics and to gain a more comprehensive picture of important determinants of an occupational safety culture in the hospital sector. In general, new and emerging occupational safety and health risks associated with digitalization should be also included in the assessment of determinants of occupational safety culture in all workplaces.

## Figures and Tables

**Table 1:** Clusters, topics and categories according to Cornelissen et al. [5].

Cluster	Topics and Categories
1. Workplace characteristics and circumstances	Physical work environment: Company size, workplace hazards, safety equipment, safety of equipment, physical workplace (design) Work characteristics: work characteristics, perceived workplace safety, goal setting, stress, shifts, working hours, job demands, job resources, production pressure, task clarity, safety control Workforce: contract type, job level, workforce quantity, workforce composition, unions, HR
2. Climate and culture	Organizational climate and culture: Organizational climate, organizational culture Safety climate and culture: safety climate, safety culture
3. Management and colleagues	Management attitudes and behaviours: leadership style, management attitudes, management behaviours, safety importance for management Co-worker attitudes and behaviours: co-worker attitudes, co-worker behaviours Management of safety: management of safety, inspections, accident analysis and record keeping, safety representations, sanctions, rewards, accident reducing measures, training, safety communication, safety policies and procedures, safety meetings and activities
4. Employee characteristics	Employee demographics: age, gender, education, disabilities, psychophysical states Career and job attitudes: tenure/experience, employee work attitudes, trust Safety characteristics: employee safety attitudes, safety motivation, safety knowledge (sharing), responsibility Lifestyle: work-life balance, marital status, children, lifestyle, lifestyle disorders and substance abuse
5. External	Governmental bodies: law and legislation, governmental bodies Stakeholders: client involvement, customer satisfaction Socio-economic: economic factors, insurance, and costs of safety
6. Performance	Safety-related performance: safety performance, PPE use, safety compliance, safety participation Organizational performance: organizational performance, financial performance, (employee) work performance, organizational quality performance, production performance, environmental performance
7. Safety outcomes	Incidents Accidents Injuries

**Table 2:** Hospital workplaces—17 studies—mapping of the investigated determinants to clusters according to Cornelisson et al. [5] (for details of the studies see Table S5 in supplementary material, for topics and categories of the clusters see Table 1)

Author and Year of Publication	(1) Workplace Characteristics and Circumstances	(2) Climate and Culture	(3) Management and Colleagues	(4) Employee Characteristics	(5) External	(6) Performance	(7) Safety Outcomes	Summary of Investigated Factors (According to Cornelissen et al. [5])
Aljabri et al. 2020 [41]	X		X	X		X	X	(1) (3) (4) (6) (7)
Bronkhorst et al. 2016 [44]	X		X	X			X	(1) (3) (4) (7)
Dal Corso 2008 [48]		X	X	X		X		(2) (3) (4) (6)
Gershon et al. 2000 [54]	X		X	X		X	X	(1) (3) (4) (6) (7)
Halbesleben et al. 2013 [56]			X			X	X	(3) (6) (7)
Manapragada et al. 2019 [62]	X		X			X		(1) (3) (6)
McCaughey et al. 2011 [63]	X		X					(1) (3)
McCaughey et al. 2013 [64]	X		X	X			X	(1) (3) (4) (7)
McCaughey et al. 2015 [65]	X		X	X			X	(1) (3) (4) (7)
McLinton et al. 2018 [66]	X		X	X			X	(1) (3) (4) (7)
McLinton et al. 2019 [67]	X		X	X			X	(1) (3) (4) (7)
Neal et al. 2000 [69]	X	X	X	X		X		(1) (2) (3) (4) (6)
Neal et al. 2006 [70]			X	X		X	X	(3) (4) (6) (7)
Nixon et al. 2015 [71]	X		X	X		X	X	(1) (3) (4) (6) (7)
Silver et al. 2019 [80]	X		X	X				(1) (3) (4)
Stone et al. 2006 [81]	X	X		X			X	(1) (2) (4) (7)
Zadow et al. 2017 [84]	X		X	X			X	(1) (3) (4) (7)
Summary of counting	14/17	3/17	16/17	14/17	0/17	8/17	12/17	

**Table 3:** Other workplaces—27 studies—mapping of the investigated determinants to clusters according to Cornelisson et al. [5] (for details of the studies see Table S5 in Supplementary Material, for topics and categories of the clusters see Table 1)

Author and Year of Publication	(1) Workplace Characteristics and Circumstances	(2) Climate and Culture	(3) Management and Colleagues	(4) Employee Characteristics	(5) External	(6) Performance	(7) Safety Outcomes	Summary of Investigated Factors (According to Cornelissen et al. [5])
Beus et al. 2010 [42]	X		X	X				(1) (3) (4)
Brondino et al. 2012 [43]	X		X	X		X		(1) (3) (4) (6)
Bunner et al. 2018 [45]	X		X	X		X		(1) (3) (4) (6)
Chen et al. 2017 [46]	X		X	X			X	(1) (3) (4) (7)
Clarke et al. 2006 [47]	X		X	X		X		(1) (3) (4) (6)
DeJoy et al. 2004 [49]	X		X	X		X		(1) 3) (4) (6)
DeJoy et al. 2010 [50]	X	X	X	X		X	X	(1) (2) (3) (4) (6) (7)
Fernández-Muñiz et al. 2007 [51]			X	X		X	X	(3) (4) (6) (7)
Fernández-Muñiz et al. 2012 [52]	X		X	X	X	X	X	(1) (3) (4) (5) (6) (7)
Garcia et al. 2004 [53]	X		X	X		X		(1) (3) (4) (6)
Griffin et al. 2000 [55]			X	X		X		(1) (3) (4) (6)
Hicks et al. 2016 [57]	X		X	X		X	X	(1) (3) (4) (6) (7)
Huang et al. 2006 [58]			X	X			X	(3) (4) (7)
Kath et al. 2010 [59]	X		X	X			X	(1) (3) (4) (7)
Katz et al. 2019 [60]	X			X		X	X	(1) (4) (6) (7)
Larsson et al. 2008 [61]	X			X		X		(1) (4) (6)
Milijić et al. 2014 [68]	X		X	X			X	(1) (3) (4) (7)
Nordlöf et al. 2017 [72]	X		X	X	X	X	X	(1) (3) (4) (5) (6) (7)
Oliver et al. 2006 [73]	X		X	X		X	X	(1) (3) (4) (6) (7)
Pandit et al. 2019 [74]	X		X	X			X	(1) (3) (4) (7)
Probst et al. 2004 [75]	X		X	X		X	X	(1) (3) (4) (6) (7)
Probst et al. 2008 [76]			X				X	(3) (7)
Probst et al. 2015 [77]			X				X	(3) (7)
Rodrigues et al. 2015 [78]	X		X	X		X		(1) (3) (4) (6)
Schwatka et al. 2016 [79]			X			X		(3) (6)
Tholén et al. 2013 [82]	X		X			X		(1) (3) (6)
Tomás et al. 2011 [83]	X		X	X		X	X	(1) (3) (4) (6) (7)
Summary of counting	21/27	1/27	25/27	23/27	2/27	19/27	16/27	

**Table 4:** Summary and classification of the studies addressing cluster, topics, and categories according to Cornelissen et al. [5]

Cluster	Categories	Studies at Hospital Workplace	Studies at other Workplaces
1. Workplace characteristics and circumstances	Physical work environment	[54,63,71,80,81]	[42,49,53,57,60,68,72,73,78,83]
	Work characteristics	[41,44,62,63,64,65,66,67,69,80,84]	[42,43,45,46,49,50,52,53,57,59,60,61,72,74,75,82]
	Workforce	[41,44,64,66,80,84]	[43,45,46,47,53,57,60,68,72,73]
2. Climate and culture	Organizational climate and culture	[48,69,81]	[50]
	Safety climate and culture		
3. Management and colleagues	Management attitudes and behaviours	[41,44,48,54,56,62,63,64,65,66,67,69,70,80,84]	[43,45,46,47,49,50,51,52,55,57,58,59,68,72,74,75,77,78,79,82,83]
	Co-worker attitudes and behaviours	[44,63,64,65]	[43,46,49,79]
	Management of safety	[44,54,62,63,64,65,66,67,69,71,80,84]	[42,43,45,46,49,50,51,52,53,55,57,58,59,68,72,73,75,76,77,78,82,83]
4. Employee characteristics	Employee demographics	[41,44,54,64,65,66,80,81,84]	[43,46,47,49,53,57,60,68,72,73,78]
	Career and job attitudes	[41,44,64,65,66,71,80,81]	[42,43,46,47,49,50,52,53,59,60,61,68,78]
	Safety characteristics	[48,65,69,70]	[45,46,51,55,59,61,68,72,73,74,75,83]
	Lifestyle	[44,67]	[50,60]
5. External	Governmental bodies		
	Stakeholders		[52]
	Socio-economic		[72]
6. Performance	Safety-related performance	[41,48,54,56,62,69,70,71]	[43,45,47,49,50,51,52,53,55,57,58,61,72,73,75,78,79,82,83]
	Organizational performance		[52,60,72]
7. Safety outcomes	Incidents	[66,67]	[46,72]
	Accidents	[70]	[50,68,73,75,77,83]
	Injuries	[41,54,56,64,65,71,81,84]	[51,52,58,59,74,75,76,77]

## Supplementary Materials

The following are available online at https://www.mdpi.com/1660-4601/17/18/6588/s1, Table S1: Modified PRISMA checklist according to Moher et al. 2009 [22]; Table S2: Example for search strategy in Pubmed: Text word search; Table S3: Example for search strategy in Pubmed: MeSH-Tetablerm search; Table S4: Quality appraisal items; Table S5: Overview of the study characteristics and mapping the clusters according to Cornelissen et al. [5].

## References

[1] Zohar D., Le Quick J.C.T. (2011). Safety climate: Conceptualization, measurement, and improvement. Handbook of Occupational Health Psychology.

[2] He Y., Wang Y., Payne S.C. (2019). How is safety climate formed? A meta-analysis of the antecedents of safety climate. Organ. Psychol. Rev..

[3] Beus J.M., McCord M.A., Zohar D. (2016). Workplace safety. Organ. Psychol. Rev..

[4] Clarke S. (2010). An integrative model of safety climate: Linking psychological climate and work attitudes to individual safety outcomes using meta-analysis. J. Occup. Organ. Psychol..

[5] Cornelissen P.A., van Hoof J.J., De Jong M.D.T. (2017). Determinants of safety outcomes and performance: A systematic literature review of research in four high-risk industries. J. Saf. Res..

[6] Halligan M., Zecevic A. (2011). Safety culture in healthcare: A review of concepts, dimensions, measures and progress. BMJ Qual. Saf..

[7] Pumar-Méndez M.J., Attree M., Wakefield A. (2014). Methodological aspects in the assessment of safety culture in the hospital setting: A review of the literature. Nurse Educ. Today.

[8] Weaver M.D., Wang H.E., Fairbanks R.J., Patterson D. (2012). The association between EMS workplace safety culture and safety outcomes. Prehosp. Emerg. Care.

[9] Taylor J.A., Dominici F., Agnew J., Gerwin D., Morlock L., Miller M.R. (2012). Do nurse and patient injuries share common antecedents? An analysis of associations with safety climate and working conditions. BMJ Qual. Saf..

[10] Wagner A., Rieger M.A., Manser T., Sturm H., Hardt J., Martus P., Lessing C., Hammer A. (2019). Healthcare professionals’ perspectives on working conditions, leadership, and safety climate: A cross-sectional study. BMC Health Serv. Res..

[11] Wagner A., Hammer A., Manser T., Martus P., Sturm H., Rieger M.A. (2018). Do Occupational and Patient Safety Culture in Hospitals Share Predictors in the Field of Psychosocial Working Conditions? Findings from a Cross-Sectional Study in German University Hospitals. Int. J. Environ. Res. Public Health.

[12] Wagner A., Michaelis M., Luntz E., Wittich A., Schrappe M., Lessing C., Rieger M.A. (2018). Assessment of Patient and Occupational Safety Culture in Hospitals: Development of a Questionnaire with Comparable Dimensions and Results of a Feasibility Study in a German University Hospital. Int. J. Environ. Res. Public Health.

[13] Sturm H., Rieger M.A., Martus P., Ueding E., Wagner A., Holderried M., Maschmann J. (2019). Do perceived working conditions and patient safety culture correlate with objective workload and patient outcomes: A cross-sectional explorative study from a German university hospital. PLoS ONE.

[14] Agnew C., Flin R., Mearns K. (2013). Patient safety climate and worker safety behaviours in acute hospitals in Scotland. J. Saf. Res..

[15] Trinkoff A.M., Le R., Geiger-Brown J., Lipscomb J. (2007). Work schedule, needle use, and needlestick injuries among registered nurses. Infect. Control. Hosp. Epidemiol..

[16] Wicker S., Ludwig A.-M., Gottschalk R., Rabenau H.F. (2008). Needlestick injuries among health care workers: Occupational hazard or avoidable hazard?. Wien. Klin. Wochenschr..

[17] Cook J.M., Slade M.D., Cantley L.F., Sakr C.J. (2016). Evaluation of safety climate and employee injury rates in healthcare. Occup. Environ. Med..

[18] McVicar A. (2003). Workplace stress in nursing: A literature review. Adv. Nurs..

[19] Schwatka N.V., Hecker S., Goldenhar L.M. (2016). Defining and Measuring Safety Climate: A Review of the Construction Industry Literature. Ann. Occup. Hyg..

[20] Singh V., Verma A. (2018). A review, simple meta-analysis and future directions of safety climate research in manufacturing organizations. Int. J. Occup. Saf. Ergon..

[21] Whittemore R., Knafl K. (2005). The integrative review: Updated methodology: Methodological issues in nursing research. J. Adv. Nurs..

[22] Moher D., Liberati A., Tetzlaff J., Altman D.G., The PRISMA Group (2009). Preferred Reporting Items for Systematic Reviews and Meta-Analyses: The PRISMA Statement. PLoS Med..

[23] McGowan J., Sampson M., Salzwedel D.M., Cogo E., Foerster V., Lefebvre C. (2016). PRESS Peer Review of Electronic Search Strategies: 2015 Guideline Statement. J. Clin. Epidemiol..

[24] Cooke A., Smith D., Booth A. (2012). Beyond PICO: The SPIDER tool for qualitative evidence synthesis. Qual. Health Res..

[25] Luria G., Yagil D. (2010). Safety perception referents of permanent and temporary employees: Safety climate boundaries in the industrial workplace. Accid. Anal. Prev..

[26] Luria G. (2010). The social aspects of safety management: Trust and safety climate. Accid. Anal. Prev..

[27] Zohar D. (2000). A group-level model of safety climate: Testing the effect of group climate on microaccidents in manufacturing jobs. J. Appl. Psychol..

[28] Zohar D. (2002). The effects of leadership dimensions, safety climate, and assigned priorities on minor injuries in work groups. J. Organiz. Behav..

[29] Zohar D., Luria G. (2004). Climate as a social-cognitive construction of supervisory safety practices: Scripts as proxy of behavior patterns. J. Appl. Psychol..

[30] Zarei E., Khakzad N., Reniers G., Akbarid R. (2016). On the relationship between safety climate and occupational burnout in healthcare organizations. Saf. Sci..

[31] Khosravi Y., Asilian-Mahabadi H., Hajizadeh E., Hassanzadeh-Rangi N., Bastani H., Khavanin A., Mortazavi S.B. (2014). Modeling the Factors Affecting Unsafe Behavior in the Construction Industry from Safety Supervisors’ Perspective - PubMed. J. Res. Health Sci..

[32] Gyekye S.A. (2005). Workers’ perceptions of workplace safety and job satisfaction. Int. J. Occup. Saf. Ergon..

[33] Gyekye S.A., Salminen S. (2005). Are “good soldiers” safety conscious? An examination of the relationship between organizational citizenship behaviors and perception of workplace safety. Soc. Behav. Personal..

[34] Gyekye S.A., Salminen S. (2007). Workplace safety perceptions and perceived organizational support: Do supportive perceptions influence safety perceptions?. Int. J. Occup. Saf. Ergon..

[35] Lee S., Dalal R.S. (2016). Climate as situational strength: Safety climate strength as a cross-level moderator of the relationship between conscientiousness and safety behaviour. Eur. J. Work Organ. Psychol..

[36] Ouzzani M., Hammady H., Fedorowicz Z., Elmagarmid A. (2016). Rayyan-a web and mobile app for systematic reviews. Syst. Rev..

[37] Specialist Unit for Review Evidence Questions to Assist with the Critical Appraisal of Cross-Sectional Studies. https://www.cardiff.ac.uk/__data/assets/pdf_file/0010/1142974/SURE-CA-form-for-Cross-sectional_2018.pdf.

[38] Specialist Unit for Review Evidence Questions to Assist with the Critical Appraisal of Cohort Studies. https://www.cardiff.ac.uk/__data/assets/pdf_file/0006/1142997/SURE-CA-form-for-Cohort_2018.pdf.

[39] Hong Q.N., Pluye P., Fàbregues S., Bartlett G., Boardman F., Cargo M., Dagenais P., Gagnon M.-P., Griffiths F., Nicolau B. (2018). Mixed Methods Appraisal Tool (MMAT), Version 2018. Educ. Inf..

[40] Davies M. (2011). Concept mapping, mind mapping and argument mapping: What are the differences and do they matter?. High. Educ..

[41] Aljabri D., Vaughn A., Austin M., White L., Li Z., Naessens J., Spaulding A. (2020). An Investigation of Healthcare Worker Perception of Their Workplace Safety and Incidence of Injury. Workplace Health Saf..

[42] Beus J.M., Bergman M.E., Payne S.C. (2010). The influence of organizational tenure on safety climate strength: A first look. Accid. Anal. Prev..

[43] Brondino M., Silva S.A., Pasini M. (2012). Multilevel approach to organizational and group safety climate and safety performance: Co-workers as the missing link. Saf. Sci..

[44] Bronkhorst B., Vermeeren B. (2016). Safety climate, worker health and organizational health performance. Int. J. Workplace Health Manag..

[45] Bunner J., Prem R., Korunka C. (2018). How Work Intensification Relates to Organization-Level Safety Performance: The Mediating Roles of Safety Climate, Safety Motivation, and Safety Knowledge. Front. Psychol..

[46] Chen Y., McCabe B., Hyatt D. (2017). Impact of individual resilience and safety climate on safety performance and psychological stress of construction workers: A case study of the Ontario construction industry. J. Saf. Res..

[47] Clarke S., Ward K. (2006). The role of leader influence tactics and safety climate in engaging employees’ safety participation. Risk Anal..

[48] Dal Corso L. (2008). Mediation effects of safety climate and safety motivation on the relation between organizational climate and safety performance in the workplace. TPM.

[49] DeJoy D.M., Schaffer B.S., Wilson M.G., Vandenberg R.J., Butts M.M. (2004). Creating safer workplaces: Assessing the determinants and role of safety climate. J. Saf. Res..

[50] DeJoy D.M., Della L.J., Vandenberg R.J., Wilson M.G. (2010). Making work safer: Testing a model of social exchange and safety management. J. Saf. Res..

[51] Fernández-Muñiz B., Montes-Peón J.M., Vázquez-Ordás C.J. (2007). Safety culture: Analysis of the causal relationships between its key dimensions. J. Saf. Res..

[52] Fernández-Muñiz B., Montes-Peón J.M., Vázquez-Ordás C.J. (2012). Safety climate in OHSAS 18001-certified organisations: Antecedents and consequences of safety behaviour. Accid. Anal. Prev..

[53] Garcia A.M., Boix P., Canosa C. (2004). Why do workers behave unsafely at work? Determinants of safe work practices in industrial workers. Occup. Environ. Med..

[54] Gershon R.R., Karkashian C.D., Grosch J.W., Murphy L.R., Escamilla-Cejudo A., Flanagan P.A., Bernacki E., Kasting C., Martin L. (2000). Hospital safety climate and its relationship with safe work practices and workplace exposure incidents. Am. J. Infect. Control..

[55] Griffin M.A., Neal A. (2000). Perceptions of Safety at Work: A Framework for Linking Safety Climate to Safety Performance, Knowledge, and Motivation. J. Occup. Health Psychol..

[56] Halbesleben J.R.B., Leroy H., Dierynck B., Simons T., Savage G.T., McCaughey D., Leon M.R. (2013). Living up to safety values in health care: The effect of leader behavioral integrity on occupational safety. J. Occup. Health Psychol..

[57] Hicks G., Buttigieg D., de Cieri H. (2016). Safety climate, strain and safety outcomes. J. Manag. Organ..

[58] Huang Y.-H., Ho M., Smith G.S., Chen P.Y. (2006). Safety climate and self-reported injury: Assessing the mediating role of employee safety control. Accid. Anal. Prev..

[59] Kath L.M., Magley V.J., Marmet M. (2010). The role of organizational trust in safety climate’s influence on organizational outcomes. Accid. Anal. Prev..

[60] Katz A.S., Pronk N.P., McLellan D., Dennerlein J., Katz J.N. (2019). Perceived Workplace Health and Safety Climates: Associations with Worker Outcomes and Productivity. Am. J. Prev. Med..

[61] Larsson S., Pousette A., Törner M. (2008). Psychological climate and safety in the construction industry-mediated influence on safety behaviour. Saf. Sci..

[62] Manapragada A., Bruk-Lee V., Thompson A.H., Heron L.M. (2019). When safety climate is not enough: Examining the moderating effects of psychosocial hazards on nurse safety performance. J. Adv. Nurs..

[63] McCaughey D., McGhan G., DelliFraine J.L., Brannon S.D. (2011). Perception is reality: How patients contribute to poor workplace safety perceptions. Health Care Manag. Rev..

[64] McCaughey D., DelliFraine J.L., McGhan G., Bruning N.S. (2013). The negative effects of workplace injury and illness on workplace safety climate perceptions and health care worker outcomes. Saf. Sci..

[65] McCaughey D., DelliFraine J., Erwin C.O. (2015). Best practices to promote occupational safety and satisfaction: A comparison of three North American hospitals. Adv. Health Care Manag..

[66] McLinton S.S., Dollard M.F., Tuckey M.R. (2018). New perspectives on psychosocial safety climate in healthcare: A mixed methods approach. Saf. Sci..

[67] McLinton S.S., Afsharian A., Dollard M.F., Tuckey M.R. (2019). The dynamic interplay of physical and psychosocial safety climates in frontline healthcare. Stress Health.

[68] Milijić N., Mihajlović I., Nikolić D., Živković Ž. (2014). Multicriteria analysis of safety climate measurements at workplaces in production industries in Serbia. Int. J. Ind. Ergon..

[69] Neal A., Griffin M.A., Hart P. (2000). The impact of organizational climate on safety climate and individual behavior. Saf. Sci..

[70] Neal A., Griffin M.A. (2006). A study of the lagged relationships among safety climate, safety motivation, safety behavior, and accidents at the individual and group levels. J. Appl. Psychol..

[71] Nixon A.E., Lanz J.J., Manapragada A., Bruk-Lee V., Schantz A., Rodriguez J.F. (2015). Nurse safety: How is safety climate related to affect and attitude?. Work Stress.

[72] Nordlöf H., Wiitavaara B., Högberg H., Westerling R. (2017). A cross-sectional study of factors influencing occupational health and safety management practices in companies. Saf. Sci..

[73] Oliver A., Tomás J.M., Cheyne A. (2006). Safety climate: Its nature and predictive power. Psychol. Spain.

[74] Pandit B., Albert A., Patil Y., Al-Bayati A.J. (2019). Impact of safety climate on hazard recognition and safety risk perception. Saf. Sci..

[75] Probst T.M. (2004). Safety and insecurity: Exploring the moderating effect of organizational safety climate. J. Occup. Health Psychol..

[76] Probst T.M., Brubaker T.L., Barsotti A. (2008). Organizational injury rate underreporting: The moderating effect of organizational safety climate. J. Appl. Psychol..

[77] Probst T.M. (2015). Organizational safety climate and supervisor safety enforcement: Multilevel explorations of the causes of accident underreporting. J. Appl. Psychol..

[78] Rodrigues M.A., Arezes P.M., Leão C.P. (2015). Safety climate and its relationship with furniture companies’ safety performance and workers’ risk acceptance. Theor. Issues Ergon. Sci..

[79] Schwatka N.V., Rosecrance J.C. (2016). Safety climate and safety behaviors in the construction industry: The importance of co-workers commitment to safety. Work.

[80] Silver S.R., Boiano J.M. (2019). Differences in Safety Climate Perception by Health Care Worker, Work Schedule, and Workplace Characteristics. Am. J. Med. Qual..

[81] Stone P.W., Gershon R.R.M. (2006). Nurse work environments and occupational safety in intensive care units. Policy Polit. Nurs. Pract..

[82] Tholén S.L., Pousette A., Törner M. (2013). Causal relations between psychosocial conditions, safety climate and safety behaviour–A multi-level investigation. Saf. Sci..

[83] Tomás J.M., Cheyne A., Oliver A. (2011). The Relationship Between Safety Attitudes and Occupational Accidents. Eur. Psychol..

[84] Zadow A.J., Dollard M.F., Mclinton S.S., Lawrence P., Tuckey M.R. (2017). Psychosocial safety climate, emotional exhaustion, and work injuries in healthcare workplaces. Stress Health.

[85] EU-OSHA Foresight on New and Emerging Occupational Safety and Health Risks Associated with Digitalisation by 2025: European Risk Observatory Report. https://osha.europa.eu/sites/default/files/publications/documents/Foresight_new_OSH_risks_2025_report.pdf.

[86] McCaughey D., Halbesleben J.R.B., Savage G.T., Simons T., McGhan G.E. (2013). Safety leadership: Extending workplace safety climate best practices across health care workforces. Adv. Health Care Manag..

[87] Christian M.S., Bradley J.C., Wallace J.C., Burke M.J. (2009). Workplace safety: A meta-analysis of the roles of person and situation factors. J. Appl. Psychol..

