# Supplementary material for: Determinants of Occupational Safety Culture in Hospitals and other Workplaces—Results from an Integrative Literature Review
Source: Int J Environ Res Public Health. 2020 Sep 10;17(18):6588. doi: 10.3390/ijerph17186588 (PMC7559364; doi:10.3390/ijerph17186588)
Supplement: Supplementary file 1 [file ijerph-17-06588-s001.pdf]

International Journal of
Environmental Research and Public Health

Review

Determinants of occupational safety culture in hospitals and other workplaces – results from an integrative literature review

Anke Wagner, Ladina Schöne and Monika A. Rieger

Supplementary Material

Content of Supplementary Material

Table S1: Modified PRISMA checklist according to Moher et al. 2009 [22]
Table S2: Example for search strategy in Pubmed: Text word search
Table S3: Example for search strategy in Pubmed: MeSH-Term search
Table S4: Quality appraisal items
Table S5: Overview of the study characteristics and mapping the clusters according to Cornelissen et al. [5]

Table S1: Modified PRISMA checklist according to Moher et al. 2009 [22]

Section/topic	Item No.	Checklist item	Section	Page
**TITLE**				
Title	1	Identify the report as a systematic review, meta-analysis, or both.	Title page	1
**ABSTRACT**				
Structured summary	2	Provide a structured summary including, as applicable: background; objectives; data sources; study eligibility criteria, participants, and interventions; study appraisal and synthesis methods; results; limitations; conclusions and implications of key findings; systematic review registration number.	Abstract	1
**INTRODUCTION**				
Rationale	3	Describe the rationale for the review in the context of what is already known.	Introduction	1-4
Objectives	4	Provide an explicit statement of questions being addressed with reference to participants, interventions, comparisons, outcomes, and study design (PICOS).	Not provided	-
**METHODS**				
Protocol and registration	5	Indicate if a review protocol exists, if and where it can be accessed (e.g., Web address), and, if available, provide registration information including registration number.	Not provided	-
Eligibility criteria	6	Specify study characteristics (e.g., PICOS, length of follow-up) and report characteristics (e.g., years considered, language, publication status) used as criteria for eligibility, giving rationale.	Methods	5
Information sources	7	Describe all information sources (e.g., databases with dates of coverage, contact with study authors to identify additional studies) in the search and date last searched.	Methods	4-5
Search	8	Present full electronic search strategy for at least one database, including any limits used, such that it could be repeated.	Supplementary Material	S2, S3
Study selection	9	State the process for selecting studies (i.e., screening, eligibility, included in systematic review, and, if applicable, included in the meta-analysis).	Results	6
Data collection process	10	Describe method of data extraction from reports (e.g., piloted forms, independently, in duplicate) and any processes for obtaining and confirming data from investigators.	Methods	6
Data items	11	List and define all variables for which data were sought (e.g., PICOS, funding sources) and any assumptions and simplifications made.	Methods and Supplementary Material	6, S5
Risk of bias in individual studies	12	Describe methods used for assessing risk of bias of individual studies (including specification of whether this was done at the study or outcome level), and how this information is to be used in any data	Not provided	-

		synthesis.		
Summary measures	13	State the principal summary measures (e.g., risk ratio, difference in means).	Not provided	-
Synthesis of results	14	Describe the methods of handling data and combining results of studies, if done, including measures of consistency (e.g., I^2) for each meta-analysis.	Methods	6
Risk of bias across studies	15	Specify any assessment of risk of bias that may affect the cumulative evidence (e.g., publication bias, selective reporting within studies).	Not provided	-
Additional analyses	16	Describe methods of additional analyses (e.g., sensitivity or subgroup analyses, meta-regression), if done, indicating which were pre-specified.	Not provided	-
**RESULTS**				
Study selection	17	Give numbers of studies screened, assessed for eligibility, and included in the review, with reasons for exclusions at each stage, ideally with a flow diagram.	Results	7
Study characteristics	18	For each study, present characteristics for which data were extracted (e.g., study size, PICOS, follow-up period) and provide the citations.	Results and Supplementary Material	8, S5
Risk of bias within studies	19	Present data on risk of bias of each study and, if available, any outcome level assessment (see item 12).	Not provided	-
Results of individual studies	20	For all outcomes considered (benefits or harms), present, for each study: (a) simple summary data for each intervention group (b) effect estimates and confidence intervals, ideally with a forest plot.	Not provided	-
Synthesis of results	21	Present results of each meta-analysis done, including confidence intervals and measures of consistency.	Not provided	-
Risk of bias across studies	22	Present results of any assessment of risk of bias across studies (see Item 15).	Not provided	-
Additional analysis	23	Give results of additional analyses, if done (e.g., sensitivity or subgroup analyses, meta-regression [see Item 16]).	Not provided	
**DISCUSSION**				
Summary of evidence	24	Summarize the main findings including the strength of evidence for each main outcome; consider their relevance to key groups (e.g., healthcare providers, users, and policy makers).	Discussion	14-17
Limitations	25	Discuss limitations at study and outcome levels (e.g., risk of bias), and at review-level (e.g., incomplete retrieval of identified research, reporting bias).	Discussion	17

Conclusions	26	Provide a general interpretation of the results in the context of other evidence, and implications for future research.	Discussion and Conclusion	17-18
**FUNDING**				
Funding	27	Describe sources of funding for the systematic review and other support (e.g., supply of data); role of funders for the systematic review.	Funding	18

Table S2: Example for search strategy in Pubmed: Text word search

Domain	No	Search terms
Safety culture	#1	"prevention culture" [tw]
	#2	"safety culture" [tw] OR "culture of safety" [tw]
	#3	"safety climate" [tw]
	#4	"organizational culture" [tw] OR "organizational climate" [tw]
	#5	#1 OR #2 OR #3 OR #4:<br>"prevention culture" [tw] OR "safety culture" [tw] OR "culture of safety" [tw] OR "safety climate" [tw] OR "organizational culture" [tw] OR "organizational climate" [tw]
Occupational health	#6	"occupational health" [tw] OR "occupational safety" [tw] OR "occupational health and safety" [tw]
	#7	"industrial safety" [tw] OR "on the job-safety" [tw] OR "working safety" [tw] OR "safety at work" [tw]
General workplace	#8	"workplace [tw] OR "working condition" [tw] OR "work environment" [tw]
Hospital	#9	"hospital" [tw]
Combination: Safety culture AND Occupational health AND General workplace	#10	#5 AND (#6 OR #7) AND #8:<br>("prevention culture"[tw] OR "safety culture"[tw] OR "culture of safety"[tw] OR "safety climate"[tw] OR "organizational culture"[tw] OR "organizational climate"[tw]) AND (("occupational health"[tw] OR "occupational safety"[tw] OR "occupational health and safety"[tw]) OR ("industrial safety"[tw] OR "on the job-safety"[tw] OR "working safety"[tw] OR "safety at work"[tw])) AND ("workplace"[tw] OR "working condition"[tw] OR "work environment" [tw])
Combination: Safety culture AND Occupational health AND Hospital	#11	#5 AND (#6 OR #7) AND #9:<br>("prevention culture"[tw] OR "safety culture"[tw] OR "culture of safety"[tw] OR "safety climate"[tw] OR "organizational culture"[tw] OR "organizational climate"[tw]) AND (("occupational health"[tw] OR "occupational safety"[tw] OR "occupational health and safety"[tw]) OR ("industrial safety"[tw] OR "on the job-safety"[tw] OR "working safety"[tw] OR "safety at work"[tw])) AND ("hospital"[tw])

Table S3: Example for search strategy in Pubmed: MeSH-Term search

Domain	No	Search terms
Safety culture	#1	"Safety Management" [MESH]
	#2	"Organizational Culture " [MESH]
	#3	#1 OR #2:<br>"Safety Management" [MESH] OR "Organizational Culture " [MESH]
Occupational health	#4	"Occupational Health" [MESH]
	#5	"Occupational Health Services" [MESH]
	#6	“United States Occupational Safety and Health Administration" [Mesh]
	#7	#4 OR #5 OR #6:<br>"Occupational Health" [MESH] OR "Occupational Health Services" [MESH] OR “United States Occupational Safety and Health Administration" [Mesh]
General workplace	#8	"Workplace"[MeSH] OR "Work Engagement" [MeSH]
Hospital	#9	"Hospitals"[MeSH] OR "Personnel, Hospital" [MeSH] OR "Attitude of Health Personnel"[MeSH] OR "Academic Medical Centers"[MeSH]
Combination: Safety culture AND Occupational health AND General workplace	#10	#3 AND #7 AND #8:<br>("Safety Management"[MeSH] OR "Organizational Culture"[MeSH]) AND ("Occupational Health"[MESH] OR "Occupational Health Services"[MESH] OR "United States Occupational Safety and Health Administration"[Mesh]) AND ("Workplace"[MeSH] OR "Work Engagement"[MeSH])
Combination: Safety culture AND Occupational health AND Hospital	#11	#3 AND #7 AND #9:<br>("Safety Management"[MeSH] OR "Organizational Culture"[MeSH]) AND ("Occupational Health"[MeSH] OR "Occupational Health Services"[MeSH] OR "United States Occupational Safety and Health Administration"[MeSh]) AND ("Hospitals"[MeSH] OR "Personnel, Hospital"[MeSH] OR "Attitude of Health Personnel"[MeSH] OR "Academic Medical Centers"[MeSH])

Table S4: Quality appraisal items

**Questions for the quality appraisal of cross-sectional studies (SURE criteria for cross-sectional studies, version 2018)**	
**No.**	**Items**
1	Is the study design clearly stated?
2	Does the study address a clearly focused question? Consider: Population; Exposure (defined and accurately measured?); Outcomes.
3	Are the setting, locations and relevant dates provided? Consider: recruitment period; exposure; data collection.
4	Were participants fairly selected? Consider: eligibility criteria; sources & selection of participants.
5	Are participant characteristics provided? Consider if: sufficient details; a table is included.
6	Are the measures of exposures & outcomes appropriate? Consider if the methods of assessment are valid & reliable.
7	Is there a description of how the study size was arrived at?
8	Are the statistical methods well described? Consider: How missing data was handled; were potential sources of bias (confounding factors) considered/controlled for.
9	Is information provided on participant eligibility? Consider if following provided: number potentially eligible, confirmed eligible, entered into study
10	Are the results well described? Consider if: effect sizes, confidence intervals/standard deviations provided; the conclusions are the same in the abstract and the full text.
11	Is any sponsorship/conflict of interest reported?
12	Finally: Did the authors identify any limitations and, if so, are they captured above?
**Questions for the quality appraisal of cohort studies (SURE criteria for cohort studies, version 2018)**	
**No.**	**Items**
1	Is the study design clearly stated?
2	Does the study address a clearly focused question? Consider: Population; Exposure (defined and accurately measured?); Comparator/Control; Outcomes.
3	Are the setting, locations and relevant dates provided? Consider: recruitment period; exposure; follow-up & data collection.
4	Were participants fairly selected? Consider: eligibility criteria; sources & selection of participants; method of follow-up; for matched studies – details of matching criteria and number of exposed or unexposed.
5	Are participant characteristics provided? Consider if: sufficient details; a baseline table is included.
6	Are the measures of exposures & outcomes appropriate? Consider if the methods of assessment are valid & reliable.
7	Was bias considered? e.g. recall or selection bias
8	Is there a description of how the study size was arrived at?

9	Are the statistical methods well described? Consider: How missing data was handled; were potential sources of bias (confounding factors) controlled for; How loss to follow-up was addressed.
10	Is information provided on participant flow? Consider if following provided: flow diagram; numbers of participants at each stage; details of drop-outs; details of missing participant data; follow-up time summarised; numbers of outcome events.
11	Are the results well described? Consider if: effect sizes, confidence intervals/standard deviations provided; the conclusions are the same in the abstract and the full text.
12	Is any sponsorship/conflict of interest reported?
13	Finally...Did the authors identify any limitations and, if so, are they captured above?
**Questions for the quality appraisal of mixed-methods studies (Mixed Methods Appraisal Tool (MMAT), version 2018)**	
**No.**	**Items**
1	Is there an adequate rationale for using a mixed methods design to address the research question?
2	Are the different components of the study effectively integrated to answer the research question?
3	Are the outputs of the integration of qualitative and quantitative components adequately interpreted?.
4	Are divergences and inconsistencies between quantitative and qualitative results adequately addressed?
5	Do the different components of the study adhere to the quality criteria of each tradition of the methods involved?

Table S5: Overview of the study characteristics and mapping the clusters according to Cornelissen et al. [5]

Author and year of publication	Workplace / sector	Country	Design and data collection methods	Sample (n=)	Assessment of variables	Mapping to the criteria according to Cornelissen et al. 2017	Quality rating (Percentage of checklist met)
Aljabri et al. 2020	Hospital sector	United States of America	Cross-sectional study: self report questionnaire, injury reports	23,599 employees across 1,805 work units	– Safety climate: Everyone takes responsibility for complying with safety rules, supervisor responds quickly when safety problems are discovered<br>– Injury reports: Employee injuries, illnesses, days missed from work, and days restricted to other duties<br>– Covariates: Work unit, age, gender, total number of employees, average length of service for employees and supervisors, job titles, job tasks and functions (direct patient care versus no direct care)	(1) Workplace characteristics and circumstances<br>Total number of employees, Work unit, job titles, job tasks and functions (direct patient care versus no direct care)<br>(3) Management and colleagues<br>Supervisor responds quickly when safety problems are discovered<br>(4) Employee characteristics<br>Age, gender, average length of service for employees and supervisors<br>(6) Performance<br>Everyone takes responsibility for complying with safety rules<br>(7) Safety outcomes<br>Employee injuries, illnesses, days missed from work, and days restricted to other duties	69.2%
Beus et al. 2010	Chemical processing and manufacturing sector	Not specified (19 countries)	Cross-sectional study: self report questionnaire	80 different worksites in one industry; employees from plant (n=5,517), research and development lab (n=531), and office (n=2,920)	– Organizational tenure: Years and months<br>– Safety climate variability (i.e. strength)<br>– Control variables: Age, average working environment risk level	(1) Workplace characteristics and circumstances<br>Average working environment risk level, work stress<br>(3) Management and colleagues<br>Safety inspections, safety communication, supervisor's effort to improve safety, supervisory action and supervisory expectation, supervisor enforcement of safety policies<br>(4) Employee characteristics<br>Organizational tenure	84.6%
Brondino et al. 2012	Metal and mechanical sector	Italy	Cross-sectional study: self report questionnaire	991 blue collar workers (five workgroups)	– Organizational safety climate: Safety communication, safety	(1) Workplace characteristics and circumstances	61.5%

Table S5: Overview of the study characteristics and mapping the clusters according to Cornelissen et al. [5]

Author and year of publication	Workplace / sector	Country	Design and data collection methods	Sample (n=)	Assessment of variables	Mapping to the criteria according to Cornelissen et al. 2017	Quality rating (Percentage of checklist met)
					training, safety systems, management values<br>– Supervisor safety climate: Reaction to workers behaviours, effort to improve safety<br>– Co-worker safety climate: Safety communication, safety mentoring, safety systems, co-worker values<br>– Safety performance: Safety compliance, safety participation<br>– Background information: Gender, age, educational level, nationality, length of employment, kind of job contract, department, work shift	Job contract, department, work shift<br>(3) Management and colleagues Safety communication, safety training, safety systems, management values, supervisor safety climate (reaction to workers behaviours, effort to improve safety), co-workers' safety climate (safety communication, safety mentoring, safety systems, co-worker values)<br>(4) Employee characteristics Gender, age, educational level, nationality, length of employment<br>(6) Performance Safety compliance, safety participation	
Bronkhorst et al. 2016	Hospital sector and other healthcare sectors (mental care facilities, nursing homes, home healthcare organizations and disabled care organizations)	Netherlands	Cross-sectional study: Self report questionnaire	8,761 participants working in 177 health care organization	– Physical and psychosocial safety climate: Management priority given to health and safety; management commitment to health and safety; organizational communication; organizational participation and involvement; co-worker influence and group-norms<br>– Worker health outcomes: Musculoskeletal disorders; emotional exhaustion<br>– Organizational health performance outcomes: Absenteeism; presenteeism; health care utilization<br>– Control variables: Gender, age, tenure, supervisory position, patient/client contact, smoking, exercise	(1) Workplace characteristics and circumstances Supervisory position, patient/client contact<br>(3) Management and colleagues Management priority given to health and safety; management commitment to health and safety; organizational communication; organizational participation and involvement; co-worker influence and group-norms<br>(4) Employee characteristics Gender, age, tenure, smoking, exercise<br>(7) Safety outcomes	53.8%

Table S5: Overview of the study characteristics and mapping the clusters according to Cornelissen et al. [5]

Author and year of publication	Workplace / sector	Country	Design and data collection methods	Sample (n=)	Assessment of variables	Mapping to the criteria according to Cornelissen et al. 2017	Quality rating (Percentage of checklist met)
						Musculoskeletal disorders, emotional exhaustion, absenteeism; presenteeism; health care utilization	
Bunner et al. 2018	Manufacturing, construction, trade and maintenance, traffic and warehousing, agriculture, forestry, fishery, water supply, sewage and waste disposal, removal of pollution, other sectors	Austria	Cross-sectional study: Self report questionnaire	122 high-risk organizations (safety engineers and managers as one representative per organization)	– Work intensification: Job demands<br>– Safety climate: Management values, safety practices, safety communication, safety training and safety equipment<br>– Safety motivation and safety knowledge<br>– Safety compliance and safety participation<br>– Control variables: Number of employees, respondents' role	(1) Workplace characteristics and circumstances<br>Job demands, number of employees, respondents' role<br>(3) Management and colleagues<br>Management values, safety practices, safety communication, safety training<br>(4) Employee characteristics<br>Safety motivation, safety knowledge<br>(6) Performance<br>Safety compliance, safety participation, safety equipment	76.9%
Chen et al. 2017	Construction sector	Canada	Cross-sectional study: Self report questionnaire	837 workers from 112 construction sites	– Individual resilience<br>– Safety climate: Management commitment to safety, supervisor safety perception, co-worker safety perception, role overload, work pressure, safety knowledge<br>– Incident reporting: Physical symptoms, unsafe events, and psychological stress symptoms<br>– Demographic section: Age, tenure, gender, weekly working hours, union member, accomplished safety training, current job position	(1) Workplace characteristics and circumstances<br>Role overload, work pressure, weekly working hours, union member, current job position<br>(3) Management and colleagues<br>Management commitment to safety, supervisor safety perception, co-worker safety perception, safety training<br>(4) Employee characteristics<br>Individual resilience, safety knowledge, age, tenure, gender<br>(7) Safety outcomes<br>Physical symptoms, unsafe events, psychological stress symptoms	69.2%

Table S5: Overview of the study characteristics and mapping the clusters according to Cornelissen et al. [5]

Author and year of publication	Workplace / sector	Country	Design and data collection methods	Sample (n=)	Assessment of variables	Mapping to the criteria according to Cornelissen et al. 2017	Quality rating (Percentage of checklist met)
Clarke et al. 2006	Manufacturing sector	United Kingdom	Cross-sectional study: Self report questionnaire	105 participants from two sites	– Safety climate: Supervisory action, supervisory expectation<br>– Leader influence tactics: Pressure, upward appeals, exchange, coalition, ingratiation, rational persuasion, inspirational appeals, consultation<br>– Safety participation<br>– Demographics: Gender, age, tenure, job position	(1) Workplace characteristics and circumstances<br>Job position<br>(3) Management and colleagues<br>Supervisory action, supervisory expectation, leader influence tactics<br>(4) Employee characteristics<br>Gender, age, tenure<br>(6) Performance<br>Safety participation	69.2%
Dal Corso 2008	Hospital sector	Italy	Cross-sectional study: Self report questionnaire	231 nursing coordinators and nurses from two hospitals	– Organizational climate: Affective, cognitive, and instrumental factor<br>– Safety climate: Manager values<br>– Safety motivation<br>– Safety performance: Safety compliance and safety participation	(2) Climate and culture<br>Affective, cognitive, and instrumental factor<br>(3) Management and colleagues<br>Manager values<br>(4) Employee characteristics<br>Safety motivation<br>(6) Performance<br>Safety compliance, safety participation	53.8%
DeJoy et al. 2004	Retail chain sector	United States of America	Cross-sectional study: Self report questionnaire (part of a larger study)	2,208 employees from 21 retail units	– Organizational climate: Organizational support, co-worker support, participation with others and with supervisors, safety communication<br>– Environmental conditions<br>– Safety policies and programs<br>– Safety climate: Management support for safety, importance of safety issues within the organization<br>– Perceived safety at work<br>– Control variables: Age, tenure, gender, number of hours worked	(1) Workplace characteristics and circumstances<br>Environmental conditions, perceived safety at work, number of hours worked<br>(3) Management and colleagues<br>Organizational support, co-worker support, safety policies and programs, safety communication, management support for safety, importance of safety issues within the organization<br>(4) Employee characteristics<br>Age, tenure, gender<br>(6) Performance<br>Participation with others and with supervisors	69.2%

Table S5: Overview of the study characteristics and mapping the clusters according to Cornelissen et al. [5]

Author and year of publication	Workplace / sector	Country	Design and data collection methods	Sample (n=)	Assessment of variables	Mapping to the criteria according to Cornelissen et al. 2017	Quality rating (Percentage of checklist met)
DeJoy et al. 2010	Retail chain sector	United States of America	Cross-sectional study: Self report questionnaire (part of a larger study)	1,723 employees from 21 retail units	– Organizational core values<br>– Occupational safety and health policies and programs<br>– Perceived organizational support<br>– Safety climate: Management support for safety, importance of safety issues within the organization<br>– Organizational commitment<br>– Withdrawal behaviours: Turnover intention, absence, lateness<br>– Vitality<br>– Perceived safety at work<br>– Accidents	(1) Workplace characteristics and circumstances<br>Perceived safety at work<br>(2) Climate and culture<br>Organizational core values<br>(3) Management and colleagues<br>Occupational safety and health policies and programs, perceived organizational support, management support for safety, importance of safety issues within the organization<br>(4) Employee characteristics<br>Turnover intention, vitality<br>(6) Performance<br>Organizational commitment<br>(7) Safety outcomes<br>Accidents, absence	76.9%
Fernández-Muñiz et al. 2007	Construction, industrial and service sectors	Spain	Cross-sectional study: Self report questionnaire	455 employees (safety officer or safety coordinator) from construction, industrial and service sectors	– Safety Management System (SMS): Safety policy, incentives, training, communication, planning (preventive, emergency), control (internal, benchmarking techniques)<br>– Managers' commitment: Managers' attitudes, managers' behaviour<br>– Employees' involvement<br>– Safety performance: Number of personal injuries, material damage, employee motivation, absenteeism, or lost time	(3) Management and colleagues<br>Safety policy, incentives, training, communication, planning (preventive, emergency), control (internal, benchmarking techniques), managers' attitudes, managers' behaviour<br>(4) Employee characteristics<br>Employee motivation<br>(6) Performance<br>Employee involvement<br>(7) Safety outcomes<br>Number of personal injuries, material damage, absenteeism, or lost time	84.6%
Fernández-Muñiz et al. 2012	Industry, construction, services, agriculture & mining sector	Spain	Cross-sectional study: Self report questionnaire	131 safety officers from different sectors (industry, construction, services, agriculture & mining)	– Management commitment<br>– Incentives<br>– Work pressure<br>– Communication	(1) Workplace characteristics and circumstances<br>Work pressure<br>(3) Management and colleagues	76.9%

Table S5: Overview of the study characteristics and mapping the clusters according to Cornelissen et al. [5]

Author and year of publication	Workplace / sector	Country	Design and data collection methods	Sample (n=)	Assessment of variables	Mapping to the criteria according to Cornelissen et al. 2017	Quality rating (Percentage of checklist met)
					– Safety behaviour: Safety compliance, safety participation<br>– Performance: Safety performance (injuries to workers, material damage), employee satisfaction (satisfaction, absenteeism, worker complaints, worker quitting), firm competitiveness (product quality, productivity, customer satisfaction, image and reputation, innovation)	Management's commitment, incentives, communication<br>(4) Employee characteristics<br>Satisfaction, worker complaints, workers quitting<br>(5) External<br>Customer satisfaction<br>(6) Performance<br>Safety compliance, safety participation, product quality, productivity, image and reputation, innovation<br>(7) Safety outcomes<br>Injuries to workers, material damage, absenteeism	
Garcia et al. 2004	Pottery sector	Spain	Cross-sectional study: Self report questionnaire	734 production workers from a pottery industry	– Safety climate: workers' health and safety are sufficiently protected, management involved in occupational risk prevention, productivity and safety at work are equally important, investment in risk prevention, supervisors encourage safety behaviour/take into account my opinion and suggestions, persons devoted to health and safety, rules for safe working, received adequate health and safety training, received adequate equipment for personal protection<br>– Worker behaviour related to health and safety<br>– Worker health and safety training<br>– Number of employees, job category, type of employment, time working in pottery industry<br>– Personal variables: Age, gender, education, children, nationality	(1) Workplace characteristics and circumstances<br>Number of employees, job category, type of employment, productivity, and safety at work are equally important, received adequate equipment for personal protection<br>(3) Management and colleagues<br>Worker health and safety training, management involved in occupational risk prevention, investment in risk prevention, supervisors encourage safety behaviour/take into account my opinion and suggestions, persons devoted to health and safety, rules for safe working, received adequate health and safety training<br>(4) Employee characteristics	84.6%

Table S5: Overview of the study characteristics and mapping the clusters according to Cornelissen et al. [5]

Author and year of publication	Workplace / sector	Country	Design and data collection methods	Sample (n=)	Assessment of variables	Mapping to the criteria according to Cornelissen et al. 2017	Quality rating (Percentage of checklist met)
						Age, gender, education, children, nationality, time working in pottery industry<br>(6) Performance<br>Worker behaviour related to health and safety	
Gershon et al. 2000	Hospital sector	United States of America	Cross-sectional study: Self report questionnaire	789 employees (nursing, technician, physician)	– Safety climate: Safety program elements, support for safety program; senior management support for safety; communication and feedback about safety; accountability and responsibility; accessibility, availability, and quantity of safety equipment, supplies and engineering controls; design, maintenance, and housekeeping of the work site; training and education; absence of job hindrances to safety<br>– Self-reported compliance rates<br>– Exposure history: Needlestick injuries; splashes to eyes or mouth; contacts with open wounds; and cuts with sharps objects<br>– Demographics: Age, sex, education, occupation, work schedule, supervisory status	(1) Workplace characteristics and circumstances<br>Occupation, work schedule, supervisory status, accessibility, availability, and quantity of safety equipment, design, maintenance, and housekeeping of the work site absence of job hindrances to safety, supplies and engineering controls<br>(3) Management and colleagues<br>Safety program elements, support for safety program; senior management support for safety; communication and feedback about safety, training, and education<br>(4) Employee characteristics<br>Age, sex, education, accountability, and responsibility<br>(6) Performance<br>Self-reported compliance rates,<br>(7) Safety outcomes<br>Needlestick injuries; splashes to eyes or mouth; contacts with open wounds; and cuts with sharps objects	84.6%
Griffin et al. 2000	Study 1: Manufacturing and mining sector	Australia	Cross-sectional study: Self report questionnaire	Study 1: 1,403 employees in seven organizations	Study 1:<br>– Safety climate: Manager values, safety inspections, personnel training, safety communication	(3) Management and colleagues<br>Manager values, safety inspections, safety practices,	53.8%

Table S5: Overview of the study characteristics and mapping the clusters according to Cornelissen et al. [5]

Author and year of publication	Workplace / sector	Country	Design and data collection methods	Sample (n=)	Assessment of variables	Mapping to the criteria according to Cornelissen et al. 2017	Quality rating (Percentage of checklist met)
	Study 2: Manufacturing sector			Study 2: 381 employees in three organizations	– Safety knowledge<br>– Safety performance: Safety compliance, and safety participation<br>Study 2:<br>– Safety climate: Manager values, safety practices, personnel training, safety communication, safety equipment<br>– Safety knowledge<br>– Compliance motivation<br>– Participation motivation<br>– Safety performance: Safety compliance, and safety participation	personnel training, safety communication<br>(4) Employee characteristics Safety knowledge, compliance motivation, participation motivation<br>(6) Performance Safety compliance, safety participation, safety equipment	
Halbesleben et al. 2013	Hospital sector	United States of America	Cohort study: Self report questionnaire at three points, organizational data (number of injuries, number of sick leave days)	658 registered nurses from four acute-care hospitals	– Behavioural integrity for safety<br>– Psychological safety toward one's supervisor<br>– Safety compliance<br>– Occupational safety: Injuries and reporting	(3) Management and colleagues Psychological safety toward one's supervisor, behavioural integrity for safety<br>(6) Performance Safety compliance<br>(7) Safety outcomes Injuries and reporting	69.2%
Hicks et al. 2016	Electricity sector	Australia	Cross-sectional study: Self report questionnaire	739 employees from seven organizations	– Safety climate: Safety management, safety standards, safety communication<br>– Emotional exhaustion<br>– Safety-related behaviour: Safety compliance<br>– Safety involvement: Safety-specific behaviour and safety involvement<br>– Control variables: Gender, age, role status, work environment, employment status, geographical location	(1) Workplace characteristics and circumstances Role status, work environment, employment status, geographical location<br>(3) Management and colleagues Safety management, safety standards, safety communication<br>(4) Employee characteristics Gender, age<br>(6) Performance Safety compliance, safety-specific behaviour, safety involvement<br>(7) Safety outcomes Emotional exhaustion	69.2%

Table S5: Overview of the study characteristics and mapping the clusters according to Cornelissen et al. [5]

Author and year of publication	Workplace / sector	Country	Design and data collection methods	Sample (n=)	Assessment of variables	Mapping to the criteria according to Cornelissen et al. 2017	Quality rating (Percentage of checklist met)
Huang et al. 2006	Manufacturing, construction, service, and transportation sector	United States of America	Cross-sectional study: Self report questionnaire	2,680 employees from 18 companies	– Injury incidence<br>– Management commitment to safety<br>– Return-to-work policies<br>– Post-injury administration<br>– Safety training<br>– Employee safety control	(3) Management and colleagues<br>Management commitment to safety, return-to-work policies, post-injury administration, safety training<br>(4) Employee characteristics<br>Employee safety control<br>(7) Safety outcomes<br>Injury incidence	69.2%
Kath et al. 2010	Grocery store chain sector	United States of America	Cross-sectional study: Self report questionnaire; organizational data	599 employees from 97 unique workgroups across the organization	– Group upward safety communication<br>– Group management attitudes toward safety<br>– Organizational trust<br>– Safety motivation<br>– Job satisfaction<br>– Intent to turnover<br>– Job safety relevance<br>– Injuries	(1) Workplace characteristics and circumstances<br>Job safety relevance<br>(3) Management and colleagues<br>Group upward safety communication, group management attitudes toward safety<br>(4) Employee characteristics<br>Safety motivation, job satisfaction, intent to turnover, organizational trust<br>(7) Safety outcomes<br>Injuries	76.9%
Katz et al. 2019	Manufacturing sector	United States of America	Cross-sectional study: Self report questionnaire	904 employees from three companies	– Conditions of work: Workplace climate (Overall, how well do you think your workplace promotes your overall health and well-being? Overall, how safe do you think your workplace is?)<br>– Worker health behaviours: Tobacco, alcohol, emotional or physical abuse, physical activity, nutrition, sleep<br>– Worker outcomes: General health, back pain, depression, job satisfaction, life satisfaction<br>– Employee outcomes: Self-reported productivity (work time missed because of health-related and non	(1) Workplace characteristics and circumstances<br>Overall, how well do you think your workplace promotes your overall health and well-being? Overall, how safe do you think your workplace is? Work limitations (physical demands, time management, productivity (Number of hours worked), job type<br>(4) Employee characteristics<br>Age, sex, education, tobacco, alcohol, emotional or physical abuse, physical	92.3%

Table S5: Overview of the study characteristics and mapping the clusters according to Cornelissen et al. [5]

Author and year of publication	Workplace / sector	Country	Design and data collection methods	Sample (n=)	Assessment of variables	Mapping to the criteria according to Cornelissen et al. 2017	Quality rating (Percentage of checklist met)
					health-related issues, number of hours worked, person's health affects work productivity and regularly scheduled activities), work limitations (physical demands, time management, mental-interpersonal, and work output)<br>– Age, sex, job type, education	activity, nutrition, sleep, job satisfaction, life satisfaction, general health<br>(6) Performance<br>Self-reported productivity (Person's health affects work productivity and regularly scheduled activities, mental-interpersonal, and work output)<br>(7) Safety outcomes<br>Self-reported productivity (Work time missed because of health-related and non health-related issues), back pain, depression	
Larsson et al. 2008	Construction industry	Sweden	Cross-sectional study: Self report questionnaire	189 non-managerial construction workers	– Psychological climate: Role clarity, influence at work, possibilities for development, predictability, sense of community, social support, feedback at work, quality of leadership<br>– Job satisfaction<br>– Workplace commitment<br>– Safety motivation<br>– Safety knowledge<br>– Self-reported safety behaviour: Structural safety behaviour, interactive safety behaviour, and personal safety behaviour	(1) Workplace characteristics and circumstances<br>Role clarity, influence at work, possibilities for development, predictability, sense of community, social support, feedback at work, quality of leadership<br>(4) Employee characteristics<br>Job satisfaction, safety motivation, safety knowledge<br>(6) Performance<br>Workplace commitment, structural safety behaviour, interactive safety behaviour, and personal safety behaviour	76.9%
Manapragada et al. 2019	Hospital sector	United States of America	Cross-sectional study: Self report questionnaire	146 nurses	– Safety communication<br>– Management values<br>– Safety systems<br>– Safety performance: safety compliance and safety participation<br>– Conflict with other nurses<br>– Lack of support	(1) Workplace characteristics and circumstances<br>Workload, conflict with other nurses, lack of support<br>(3) Management and colleagues	92.3%

Table S5: Overview of the study characteristics and mapping the clusters according to Cornelissen et al. [5]

Author and year of publication	Workplace / sector	Country	Design and data collection methods	Sample (n=)	Assessment of variables	Mapping to the criteria according to Cornelissen et al. 2017	Quality rating (Percentage of checklist met)
					– Workload	Safety communication, Management values, Safety systems<br>(6) Performance<br>Safety performance (safety compliance and safety participation)	
McCaughey et al. 2011	Hospital sector	Canada	Cross-sectional study: Self report questionnaire	218 health care providers (nurses, health care aides, allied health professionals)	– High-risk patient index: Obese, infectious disease, cognitively impaired patients<br>– Work safety: Job safety, coworker safety, supervisor safety, management safety practices, and satisfaction with safety programs<br>– Job stress	(1) Workplace characteristics and circumstances<br>Obese, infectious disease, cognitively impaired patients, job safety, job stress<br>(3) Management and colleagues<br>Coworker safety, supervisor safety, management safety practices, and satisfaction with safety programs	76.9%
McCaughey et al. 2013	Hospital sector	Canada	Cross-sectional study: Self report questionnaire	218 health care providers (nurses, health care aides, allied health professionals)	– Workplace injuries<br>– Sick days resulting from workplace injuries or work-derived illnesses<br>– Workplace safety climate perceptions: Job safety, coworker safety, supervisor safety, management safety practices, satisfaction with the safety program<br>– Job stress<br>– Job satisfaction<br>– Turnover intentions<br>– Control variables: Age, years of experience, education, employment status (full time/part time)	(1) Workplace characteristics and circumstances<br>Job safety, job stress, employment status (full time/part time)<br>(3) Management and colleagues<br>Coworker safety, supervisor safety, management safety practices, satisfaction with the safety program<br>(4) Employee characteristics<br>Job satisfaction, turnover intentions, age, years of experience, education<br>(7) Safety outcomes<br>Workplace injuries, sick days resulting from workplace injuries or work-derived illnesses	61.5%
McCaughey et al. 2015	Hospital sector	Canada / United States of America	Cross-sectional study: Self report questionnaire	352 support services workers from three acute-care hospitals	– Supervisor safety leadership<br>– Organization safety leadership<br>– Safety training<br>– Supervisor support	(1) Workplace characteristics and circumstances<br>Unit safety grade, individual safety perceptions	76.9%

Table S5: Overview of the study characteristics and mapping the clusters according to Cornelissen et al. [5]

Author and year of publication	Workplace / sector	Country	Design and data collection methods	Sample (n=)	Assessment of variables	Mapping to the criteria according to Cornelissen et al. 2017	Quality rating (Percentage of checklist met)
					– Coworker support<br>– Individual safety perceptions<br>– Unit safety grade<br>– Job satisfaction<br>– Turnover intention<br>– Employee injuries<br>– Control variables: Years of experience, age, gender, education level, and overall self-rated health status	(3) Management and colleagues<br>Supervisor safety leadership, organization safety leadership, safety training, supervisor support, coworker support<br>(4) Employee characteristics<br>Job satisfaction, turnover intention, years of experience, age, gender, education level, overall self-rated health status<br>(7) Safety outcomes<br>Employee injuries	
McLinton et al. 2018	Hospital sector	Australia	Mixed-methods study: qualitative interviews and quantitative data	27 employees (nurses, physicians, allied health professionals, and corporate services and other staff) from three hospitals	Qualitative data:<br>– Psychological safety of workers valued by the organization<br>– Management practices<br>– Staff safety incident<br>Questionnaire:<br>– Demographics: Age, length of employment, job role, how long reported to current manager<br>– Psychosocial safety climate score: Management commitment, management priority, organizational communication, organizational participation	(1) Workplace characteristics and circumstances<br>Job role, psychological safety of workers valued by the organization<br>(3) Management and colleagues<br>Management practices, management commitment, management priority, organizational communication, organizational participation, how long reported to current manager<br>(4) Employee characteristics<br>Age, length of employment<br>(7) Safety outcomes<br>Staff safety incident	60.0%
McLinton et al. 2019	Hospital sector	Australia	Longitudinal study: Self report questionnaire and objective data	436 workers (nurses, medical doctors, allied health, and management and administrative staff)	Questionnaire<br>– Psychosocial safety climate: Management commitment, organizational communication, management priority, and organizational participation<br>– Physical safety climate: physical health	(1) Workplace characteristics and circumstances<br>Emotional demands, bullying, skills discretion, engagement (vigour, dedication, absorption)<br>(3) Management and colleagues	46.2%

Table S5: Overview of the study characteristics and mapping the clusters according to Cornelissen et al. [5]

Author and year of publication	Workplace / sector	Country	Design and data collection methods	Sample (n=)	Assessment of variables	Mapping to the criteria according to Cornelissen et al. 2017	Quality rating (Percentage of checklist met)
					– Job demands: Emotional demands, bullying<br>– Job resources: Skills discretion<br>– Health outcomes: Burnout<br>– Motivational outcomes: Engagement (vigour, dedication, absorption)<br>Objective data<br>– Staff safety incidents, patient safety incidents, and absence in days lost	Management commitment, organizational communication, management priority, and organizational participation<br>(4) Employee characteristics<br>Physical health<br>(7) Safety outcomes<br>Burnout, staff safety incidents, patient safety incidents, and absence in days lost	
Milijić et al. 2014	Production sector (food industry, shoes manufacture, electrical construction, PVC joinery production, cosmetic industry, textile industry, recycling, cement production, furniture industry	Serbia	Cross-sectional study: Self report questionnaire	1,098 employees from nine organizations	– Safety awareness and competency<br>– Safety communication<br>– Organizational environment<br>– Management support<br>– Risk judgement and management reaction<br>– Safety precautions and accident prevention<br>– Safety training<br>– Demographics: Age, length of work experience, gender, involved in an occupational accident, level of education, type of organization, positions	(1) Workplace characteristics and circumstances<br>Organizational environment, type of organization, positions<br>(3) Management and colleagues<br>Safety communication, management support, risk judgement and management reaction, safety precautions and accident prevention, safety training<br>(4) Employee characteristics<br>Age, length of work experience, gender, level of education, safety awareness and competency<br>(7) Safety outcomes<br>Involved in an occupational accident	61.5%
Neal et al. 2000	Hospital sector	Australia	Cross-sectional study: Self report questionnaire	525 employees from 32 work groups in one hospital	– Organizational climate: Appraisal and recognition, goal congruency, role clarity, supportive leadership, participative decision-making, professional growth, professional interaction	(1) Workplace characteristics and circumstances<br>Role clarity<br>(2) Climate and culture<br>Appraisal and recognition, goal congruency, participative decision-	46.2%

Table S5: Overview of the study characteristics and mapping the clusters according to Cornelissen et al. [5]

Author and year of publication	Workplace / sector	Country	Design and data collection methods	Sample (n=)	Assessment of variables	Mapping to the criteria according to Cornelissen et al. 2017	Quality rating (Percentage of checklist met)
					– Safety climate: Management values, communication, training, and safety systems<br>– Determinants of safety performance: Knowledge about safety practices and procedures, motivation to perform safety-related activities procedures<br>– Components of safety performance: Compliance and participation	making, professional growth, professional interaction<br>(3) Management and colleagues Management values, supportive leadership communication, training, and safety systems<br>(4) Employee characteristics Knowledge about safety practices and procedures, motivation to perform safety-related activities procedures<br>(6) Performance Compliance and participation	
Neal et al. 2006	Hospital sector	Australia	Cohort study: Self report questionnaire and injury database	135 employees in the longitudinal sample (nursing, administration, technical support, social work, and medical)	– Safety climate: Safety valued by organization<br>– Safety motivation<br>– Safety behaviour: Compliance and participation<br>– Negative affectivity<br>– Accidents	(3) Management and colleagues Safety valued by organization<br>(4) Employee characteristics Safety motivation, negative affectivity<br>(6) Performance Compliance and participation<br>(7) Safety outcomes Accidents	69.2%
Nixon et al. 2015	Hospital sector	United States of America	Cross-sectional study: Self report questionnaire	326 nurses	– Psychological safety climate: Importance of safety procedures and compliance in an organization<br>– Job-related negative affect<br>– Job satisfaction<br>– Turnover intentions<br>– Safety workarounds<br>– Exposure to hazardous chemicals or infections, falls, or equipment hazards<br>– Injuries	(1) Workplace characteristics and circumstances Exposure to hazardous chemicals or infections, falls, or equipment hazards<br>(3) Management and colleagues Importance of safety procedures, safety workarounds<br>(4) Employee characteristics Job-related negative affect, job satisfaction, turnover intentions<br>(6) Performance Compliance in an organization	53.8%

Table S5: Overview of the study characteristics and mapping the clusters according to Cornelissen et al. [5]

Author and year of publication	Workplace / sector	Country	Design and data collection methods	Sample (n=)	Assessment of variables	Mapping to the criteria according to Cornelissen et al. 2017	Quality rating (Percentage of checklist met)
						(7) Safety outcomes<br>Injuries	
Nordlöf et a. 2017	Manufacturing sector	Sweden	Cross-sectional study: Self report questionnaire	280 respondents from 197 companies (managers=191, safety delegates=89)	– Occupational health and safety management practices: Instructions, written routines, safety delegates, risk assessment, collaboration for risk assessment, written action plan, action plan follow-up, reporting incidents, emergency plan, safety training, OHS policy statement, OHSAS 18001 certification, occupational health services, budget item for OHSM<br>– Safety culture: instructions, safety rules, risk acceptance, management commitment, productivity pressure, employee involvement, individual responsibility, incident reporting, no fatalism, blaming, peer feedback, safety training, communication, continuous improvements<br>– Work environment priority: instructions, physical working conditions, psychosocial working conditions, organizational functionality (roles, working hours, competence, routines), OHSM routines, communication and interaction, leadership, health<br>– Company size: number of employees<br>– Financial performance: company profitability, solvency, and creditworthiness<br>– Other measures: part of corporate group, sex, age, external training	(1) Workplace characteristics and circumstances<br>Part of corporate group, number of employees, physical working conditions, psychosocial working conditions, organizational functionality (roles, working hours, competence, routines), OHSAS 18001 certification, productivity pressure, OHSM routines, communication and interaction, leadership, health<br>(3) Management and colleagues<br>Instructions, written routines, safety delegates, risk assessment, collaboration for risk assessment, written action plan, action plan follow-up, reporting incidents, emergency plan, safety training, OHS policy statement, occupational health services, safety rules, risk acceptance, management commitment, no fatalism, blaming, peer feedback, safety training, communication, continuous improvements, external training<br>(4) Employee characteristics<br>Sex, age, individual responsibility<br>(5) External<br>Budget item for OHSM<br>(6) Performance	84.6%

Table S5: Overview of the study characteristics and mapping the clusters according to Cornelissen et al. [5]

Author and year of publication	Workplace / sector	Country	Design and data collection methods	Sample (n=)	Assessment of variables	Mapping to the criteria according to Cornelissen et al. 2017	Quality rating (Percentage of checklist met)
						Employee involvement, company profitability, solvency, and creditworthiness<br>(7) Safety outcomes<br>Reporting incidents	
Oliver et al. 2006	Chemical and metal industries, commerce and tourism, educational and health services, administration and banking, construction, and other manufacturing industries	Spain	Cross-sectional study: Self report questionnaire	510 workers from 90 different companies	– Socio-demographic data: age, sex, level of education, type of job and contract, sector, or size<br>– Accident rate: number of near misses, minor accidents, accidents resulting in up to three days off work, severe accidents resulting in more than three days off work<br>– Quality of basic working conditions: humidity, ventilation, temperature, workspace<br>– Risks checklist: common chemical, electrical, and mechanical hazards<br>– Organizational climate: indicators of the safety goals and standards of the company, safety management, communication on safety issues, personal involvement of the employees in safety issues, and individual responsibility for accidents	(1) Workplace characteristics and circumstances<br>Type of job and contract, sector or size, quality of basic working conditions (humidity, ventilation, temperature, workspace), common chemical, electrical, and mechanical hazards<br>(3) Management and colleagues<br>Indicators of the safety goals and standards of the company, safety management, communication on safety issues<br>(4) Employee characteristics<br>Age, sex, level of education, individual responsibility for accidents<br>(6) Performance<br>Personal involvement of the employees in safety issues<br>(7) Safety outcomes<br>Number of near misses, minor accidents, accidents resulting in up to three days off work, severe accidents resulting in more than three days off work	76.9%
Pandit et al. 2019	Construction sector	United States of America	Cross-sectional study: Self report questionnaire	280 workers from 57 workplaces	– Safety climate: Management commitment to safety, foreman/supervisor support for safety, project-level safety practices, work-related pressure	(1) Workplace characteristics and circumstances<br>Project-level safety practices, work-related pressure	53.8%

Table S5: Overview of the study characteristics and mapping the clusters according to Cornelissen et al. [5]

Author and year of publication	Workplace / sector	Country	Design and data collection methods	Sample (n=)	Assessment of variables	Mapping to the criteria according to Cornelissen et al. 2017	Quality rating (Percentage of checklist met)
					– Hazard recognition performance: percentage of hazards recognized<br>– Safety risk: Expected frequency of safety incidents, expected severity of safety incidents	(3) Management and colleagues Management commitment to safety, foreman/supervisor support for safety,<br>(4) Employee characteristics Hazard recognition performance<br>(7) Safety outcomes Expected frequency of safety incidents, expected severity of safety incidents	
Probst et al. 2004	Manufacturing sector	United States of America	Cross-sectional study: Self report questionnaire, routine data	136 production employees from one company	– Job insecurity<br>– Organizational safety climate: management values, safety communication, safety training, and safety systems<br>– Safety compliance<br>– Safety knowledge<br>– Accidents, injuries<br>– Other measures: employee safety and health, employee adherence to safety rules and procedures, meeting production schedules	(1) Workplace characteristics and circumstances Employee safety and health, job insecurity, meeting production schedules<br>(3) Management and colleagues Management values, safety communication, safety training, and safety systems<br>(4) Employee characteristics Safety knowledge<br>(6) Performance Safety compliance, employee adherence to safety rules and procedures<br>(7) Safety outcomes Accidents, injuries	53.8%
Probst et al. 2008	Construction sector	United States of America	Cross-sectional study: Self report questionnaire, routine data	1,390 employees from 38 companies	– Organizational safety climate: quality and quantity of safety communication within and between work crews and management<br>– Experienced versus reported illness/injury rates<br>– Recordable injury/illness rate<br>– Unreported injury/illness rate	(3) Management and colleagues Quality and quantity of safety communication within and between work crews and management<br>(7) Safety outcomes Experienced versus reported illness/injury rates, recordable injury/illness rate, unreported injury/illness rate	61.5%

Table S5: Overview of the study characteristics and mapping the clusters according to Cornelissen et al. [5]

Author and year of publication	Workplace / sector	Country	Design and data collection methods	Sample (n=)	Assessment of variables	Mapping to the criteria according to Cornelissen et al. 2017	Quality rating (Percentage of checklist met)
Probst et al. 2015	Manufacturing, construction, transportation, mining, pulp and paper processing, health care, food processing, and distribution sector	United States of America	Cross-sectional study: Self report questionnaire	1,238 employees from 33 organizations	– Safety climate: management values, safety communication, safety training, safety systems<br>– Supervisor enforcement of safety policies<br>– Accident underreporting: accidents, lost-time injuries, first-aid injuries, additionally reported versus unreported	(3) Management and colleagues<br>Management values, safety communication, safety training, safety systems, supervisor enforcement of safety policies<br>(7) Safety outcomes<br>Accident underreporting: accidents, lost-time injuries, first-aid injuries, additionally reported versus unreported	53.8%
Rodrigues et al. 2015	Furniture sector	Portugal	Cross-sectional study: Self report questionnaire and safety audit	403 workers from 14 companies	– Safety climate<br>o Demographics: age, gender, department/sector, professional activity, duration of current employment, number of years engaged in manual labour, previous involvement in work accidents<br>o organizational level: management investment, improvement of safety systems, safety communication<br>o group level: supervisor concerns related to worker safety practices, involvement in safety issues and efforts regarding rule compliance and safety protection use<br>o individual level: worker commitment to safety<br>– Risk acceptance: risk acceptance, trust, risk perception, benefit perception and emotions	(1) Workplace characteristics and circumstances<br>Risk perception, safety conditions of workplace, equipment, machinery, department/sector, professional activity<br>(3) Management and colleagues<br>Management investment, improvement of safety systems, safety communication, supervisor concerns related to worker safety practices, involvement in safety issues and efforts regarding rule compliance and safety protection use<br>(4) Employee characteristics<br>Age, gender, number of years engaged in manual labour, previous involvement in work accidents, duration of current employment, trust<br>(6) Performance<br>Risk acceptance, worker commitment to safety, safety audit and checklist (safety behaviour and procedures)	53.8%

Table S5: Overview of the study characteristics and mapping the clusters according to Cornelissen et al. [5]

Author and year of publication	Workplace / sector	Country	Design and data collection methods	Sample (n=)	Assessment of variables	Mapping to the criteria according to Cornelissen et al. 2017	Quality rating (Percentage of checklist met)
					– Safety performance: safety audit and checklist (safety conditions of workplace, equipment, machinery, safety behaviour and procedures		
Schwatka et al. 2016	Construction sector	United States of America	Cross-sectional study: Self report questionnaire	300 workers from three firms	– Safety climate: top management safety priority, commitment, and competence; top management safety empowerment; top management safety justice; supervisor safety priority, commitment, and competence; supervisor safety empowerment; supervisor safety justice; co-workers safety commitment<br>– Safety behaviours: safety compliance, safety participation	(3) Management and colleagues<br>Top management safety priority, commitment, and competence; top management safety empowerment; top management safety justice; supervisor safety priority, commitment, and competence; supervisor safety empowerment; supervisor safety justice; co-workers safety commitment<br>(6) Performance<br>Safety compliance, safety participation	69.2%
Silver et al. 2019	Healthcare sector	United States of America	Cross-sectional study: Self report questionnaire	1,0168 health care workers (physicians, dental practitioners, pharmacists, pharmacy technicians, nurses, technologists, technicians, respiratory therapists)	– Occupation, pay time, and tenure<br>– Work arrangement<br>– Work schedule and burden characteristics<br>– Workplace characteristics<br>– Safety climate: management commitment to health and safety /overall safety culture, safety precautions, ability to report injuries without fear of negative consequences, exposure to risk<br>– Worker demographics: sex, race, ethnicity, age, highest educational level attained, whether the employee was born in the United States, union membership	(1) Workplace characteristics and circumstances<br>Occupation, pay time, work arrangement, work schedule and burden characteristics, workplace characteristics, union membership, exposure to risk<br>(3) Management and colleagues<br>Management commitment to health and safety/overall safety culture, safety precautions, ability to report injuries without fear of negative consequences<br>(4) Employee characteristics<br>Tenure, sex, race, ethnicity, age, highest educational level attained, whether the	61.5%

Table S5: Overview of the study characteristics and mapping the clusters according to Cornelissen et al. [5]

Author and year of publication	Workplace / sector	Country	Design and data collection methods	Sample (n=)	Assessment of variables	Mapping to the criteria according to Cornelissen et al. 2017	Quality rating (Percentage of checklist met)
						employee was born in the United States	
Stone et al. 2006	Hospital sector	United States of America	Cross-sectional study: Self report questionnaire and administrative data	837 registered nurses from 39 intensive care units (23 hospitals)	– Organizational climate<br>– Musculoskeletal injury, blood and body fluid exposure, an injury or exposure<br>– Hospital characteristics<br>– Employee demographics: age, experience, education	(1) Workplace characteristics and circumstances<br>Hospital characteristics, blood and body fluid exposure, any exposure<br>(2) Climate and culture<br>Organizational climate<br>(4) Employee characteristics<br>Age, experience, education<br>(7) Safety outcomes<br>Musculoskeletal injury, any injury	61.5%
Tholén et al. 2013	Construction sector	Sweden	Cohort study: self report questionnaire (four measurement waves)	289 employees in 43 units	– Safety climate: management safety priority, management safety commitment, safety communication, workgroup safety involvement<br>– Psychosocial working conditions: role clarity, predictability/information, influence at work, possibilities for development, sense of community, social support, feedback, quality of leadership<br>– Safety behaviour: using available personal protection equipment, choosing safe working methods and procedures, taking no shortcuts with safety, prioritizing safety, compliance with rules and procedures	(1) Workplace characteristics and circumstances<br>Role clarity, predictability/ information, influence at work, possibilities for development, sense of community, social support, feedback, quality of leadership<br>(3) Management and colleagues<br>Management safety priority, management safety commitment, safety communication<br>(6) Performance<br>Workgroup safety involvement, using available personal protection equipment, choosing safe working methods and procedures, taking no shortcuts with safety, prioritizing safety, compliance with rules and procedures	69.2%

Table S5: Overview of the study characteristics and mapping the clusters according to Cornelissen et al. [5]

Author and year of publication	Workplace / sector	Country	Design and data collection methods	Sample (n=)	Assessment of variables	Mapping to the criteria according to Cornelissen et al. 2017	Quality rating (Percentage of checklist met)
Tomás et al. 2011	Metal, service, educational and health services, administration or banks, construction, chemical companies, manufacturing and commerce and tourism sector	Spain	Cross-sectional study: Self report questionnaire	1,234 workers	– Individual accident rates: near misses, minor accidents, accidents resulting in up to three days off, severe accidents resulting in three or more days off work<br>– Working environment: basic work environmental conditions (humidity, lighting, working space, and ventilation) and workplace hazard checklist (type of hazard and consequences)<br>– Safety climate: safety management, communication, personal involvement, individual responsibility, and individual standards of behaviour	(1) Workplace characteristics and circumstances<br>Basic work environmental conditions (humidity, lighting, working space, and ventilation) and workplace hazard checklist (type of hazard and consequences)<br>(3) Management and colleagues<br>Safety management, communication<br>(4) Employee characteristics<br>Individual responsibility<br>(6) Performance<br>Personal involvement, and individual standards of behaviour<br>(7) Safety outcomes<br>Near misses, minor accidents, accidents resulting in up to three days off, severe accidents resulting in three or more days off work	61.5%
Zadow et al. 2017	Hospital sector	Australia	Cross-sectional study: Self report questionnaire, registered data for work injuries	214 employees from 18 teams and from three hospitals	– Psychosocial safety climate: Management commitment, organizational communication, management priority, and organizational participation<br>– Physical safety climate: Management commitment, organizational communication, management priority, and organizational participation<br>– Emotional exhaustion<br>– Demographic: Age, gender, employment status, hours worked last week, position, number of workers in the team, patient care rates<br>– Self-report reported work injuries	(1) Workplace characteristics and circumstances<br>Employment status, hours worked last week, position, number of workers in the team, patient care rates<br>(3) Management and colleagues<br>Management commitment, organizational communication, management priority, organizational participation<br>(4) Employee characteristics<br>Age, gender<br>(7) Safety outcomes<br>Self-report reported work injuries, organization	61.5%

Table S5: Overview of the study characteristics and mapping the clusters according to Cornelissen et al. [5]

Author and year of publication	Workplace / sector	Country	Design and data collection methods	Sample (n=)	Assessment of variables	Mapping to the criteria according to Cornelissen et al. 2017	Quality rating (Percentage of checklist met)
					– Organization registered work injuries<br>– Underreported work injuries	registered work injuries, underreported work injuries, emotional exhaustion	
